# Identifying, understanding, and correcting technical artifacts on the sex chromosomes in next-generation sequencing data

**DOI:** 10.1093/gigascience/giz074

**Published:** 2019-07-09

**Authors:** Timothy H Webster, Madeline Couse, Bruno M Grande, Eric Karlins, Tanya N Phung, Phillip A Richmond, Whitney Whitford, Melissa A Wilson

**Affiliations:** 1 School of Life Sciences, Arizona State University, 427 E Tyler Mall, Tempe, AZ 85281, USA; 2 Department of Anthropology, University of Utah, 260 S Central Drive, Carolyn and Kem Gardner Commons, Suite 4625, Salt Lake City, UT 84112, USA; 3 University of British Columbia, 2329 West Mall, Vancouver, BC, V6T 1Z4, Canada; 4 BC Children's Hospital Research Institute, 950 W 28th Avenue, Vancouver, BC, V5Z 4H4, Canada; 5 Department of Molecular Biology and Biochemistry, Simon Fraser University, 8888 University Drive, Burnaby, BC, V5A 1S6, Canada; 6 Division of Cancer Epidemiology and Genetics, National Cancer Institute, National Institutes of Health, 9609 Medical Center Drive, MSC 9776, Bethesda, MD 20892, USA; 7 Interdepartmental Program in Bioinformatics, UCLA, 621 Charles E. Young Drive South, Los Angeles, CA 90095-1606, USA; 8 Centre for Molecular Medicine and Therapeutics, University of British Columbia, 950 West 28th Avenue, Vancouver, BC, V52 4H4, Canada; 9 School of Biological Sciences, The University of Auckland, Private Bag 92019, Auckland 1142, New Zealand; 10 Centre for Brain Research, The University of Auckland, Private Bag 92019, Auckland 1142, New Zealand; 11 Center for Evolution and Medicine, Arizona State University, 401 E. Tyler Mall, Tempe, AZ 85287, USA

**Keywords:** X chromosome, Y chromosome, ploidy, aneuploidy, genomics, variant calling, mapping

## Abstract

**Background:**

Mammalian X and Y chromosomes share a common evolutionary origin and retain regions of high sequence similarity. Similar sequence content can confound the mapping of short next-generation sequencing reads to a reference genome. It is therefore possible that the presence of both sex chromosomes in a reference genome can cause technical artifacts in genomic data and affect downstream analyses and applications. Understanding this problem is critical for medical genomics and population genomic inference.

**Results:**

Here, we characterize how sequence homology can affect analyses on the sex chromosomes and present XYalign, a new tool that (1) facilitates the inference of sex chromosome complement from next-generation sequencing data; (2) corrects erroneous read mapping on the sex chromosomes; and (3) tabulates and visualizes important metrics for quality control such as mapping quality, sequencing depth, and allele balance. We find that sequence homology affects read mapping on the sex chromosomes and this has downstream effects on variant calling. However, we show that XYalign can correct mismapping, resulting in more accurate variant calling. We also show how metrics output by XYalign can be used to identify XX and XY individuals across diverse sequencing experiments, including low- and high-coverage whole-genome sequencing, and exome sequencing. Finally, we discuss how the flexibility of the XYalign framework can be leveraged for other uses including the identification of aneuploidy on the autosomes. XYalign is available open source under the GNU General Public License (version 3).

**Conclusions:**

Sex chromsome sequence homology causes the mismapping of short reads, which in turn affects downstream analyses. XYalign provides a reproducible framework to correct mismapping and improve variant calling on the sex chromsomes.

## Introduction

Accurate genotyping and variant calling are priorities in medical genetics, including molecular diagnostics, and population genomics [1, 2]. Despite the availability of numerous powerful tools developed to infer genotypes from sequencing data, sequence homology among genomic regions still presents a major challenge to genome assembly, short-read mapping, and variant calling. Specifically, similar sequence content can confound the mapping of short next-generation sequencing reads to a reference genome and lead to technical artifacts in downstream analyses and applications. Heteromorphic sex chromosomes, in particular, present a case of sequence homology likely to affect all individuals in a given species.

Sex chromosomes in therians—the clade containing eutherian mammals and marsupials—share a common evolutionary origin as a pair of homologous autosomes [3]. Approximately 180–210 million years ago, they began differentiating from each other through a series of recombination suppression events and subsequent gene loss on the Y chromosome [4–7]. However, this pattern is not unique to mammalian evolution or even XX/XY systems and occurs often across taxa with genetic sex determination [8, 9]. This shared origin and complex history characteristic of sex chromosomes lead to unique challenges for genome assembly and analysis, including large blocks of homologous sequence between the sex chromosomes—called gametologous sequence—that we hypothesize can lead to the mismapping of reads between the sex chromosomes. Best known of these gametologous sequences are pseudoautosomal regions (PARs; of which humans have 2: PAR1 and PAR2), found in many species—regions identical in sequence between the 2 sex chromosomes that pair and recombine during meiosis in males [10–13]. A reference genome that includes the entire sequence content from both sex chromosomes will thus duplicate gametologous regions and should substantially reduce mapping quality in these regions because most reads will identically map to 2 regions in the reference assembly. This stands in contrast to autosomal sequence, for which each diploid autosome is represented just once in the reference genome. The technical challenges presented by the biological realities of the sex chromosomes might lead to erroneous genotype calls. This is unfortunate because the sex chromosomes contribute to phenotype and disease etiology (e.g., [14]) and are useful in population genetic inference of demography and patterns of natural selection [15–19].

A number of tools, methods, and frameworks have been developed to aid in the identification of sex-linked sequence (e.g., [20]), inference of an individual's sex chromosome complement (e.g., [21]), and handling of some of the technical challenges that sex chromosomes present in genome-wide association studies (e.g., [22]). However, to our knowledge, there is no tool that simultaneously facilitates the identification of sex chromosome complement and corrects for associated technical artifacts for the purposes of short-read mapping and variant calling.

Out of the urgent need to understand the effects of sex chromosome homology on next-generation sequencing analyses, in this paper we first test whether sequence homology between sex chromosomes can confound aspects of read mapping and lead to downstream errors in sequence analysis. We then present XYalign, a tool developed to perform 3 major tasks: (1) aid in the characterization of an individual's sex chromosome complement; (2) identify and correct for technical artifacts arising from sex chromosome sequence homology; and (3) tabulate and visualize important metrics for quality control such as mapping quality, sequencing depth, and allele balance. We show how XYalign can be used to identify XX and XY individuals across sequencing depths and capture techniques. We also show that the default steps taken by XYalign correct many mismapped reads on the sex chromosomes, resulting in more accurate variant calling. Finally, because XYalign is designed to be both scalable and customizable, we discuss how it can be used in a variety of situations including genetic sex identification in both XX/XY and ZZ/ZW systems, identification of sex-linked sequences and PARs in new draft genomes, correction of technical artifacts in genomic and transcriptomic data, detection of aneuploidy, and investigation of mapping success across arbitrary chromosomes.

## Software Description

### Implementation

XYalign (SciCrunch RRID:SCR_016661) is implemented in Python and uses a number of third-party Python packages including Matplotlib [23], NumPy [24], Pandas [25], PyBedTools [26, 27], PySam [28], and SciPy [29]. It further wraps the following external tools: repair.sh and shuffle.sh from BBTools [30], BWA [31], Platypus [32], Sambamba [33], and SAMtools [34].

### Modules

XYalign is composed of 6 modules that can be called individually or serve as steps in a full pipeline: PREPARE_REFERENCE, CHROM_STATS, ANALYZE_BAM, CHARACTERIZE_SEX_CHROMS, STRIP_READS, and REMAPPING. Below, we discuss each module as a step in the full XYalign pipeline using human samples (XX/XY sex determination) as an example. Note, however, that XYalign will work with other sex chromosome systems (e.g., ZZ/ZW) and on arbitrary chromosomes (e.g., detecting autosomal aneuploidy).

The PREPARE_REFERENCE module generates 2 versions of the same reference genome: 1 for the homogametic sex (e.g., XX) and 1 for the heterogametic sex (e.g., XY). In the simplest case, it will completely hard-mask the Y chromosome with Ns in the XX version of the reference. Optionally, it will also accept ≥1 BED files containing regions to hard-mask in both reference versions. If PARs are present on both sex chromosome sequences in the reference, we strongly suggest masking the PARs on the Y chromosome, allowing reads from these regions to map exclusively to the X chromosome in XY individuals. In XYalign, we use hard masks, rather than omitting the Y chromosome in the XX reference version, because these hard masks allow files from both references to share the same sequence dictionaries and indices, thus permitting seamless integration of files from both references into downstream analyses (e.g., joint variant calling).

The CHROM_STATS module provides a relatively quick comparison of mapping quality and sequencing depth across ≥1 chromosomes and over multiple BAM files. While this provides a less detailed perspective than ANALYZE_BAM or CHARACTERIZE_SEX_CHROMS (detailed below), we envision it to be especially useful in ≥2 different cases. First, in well-characterized systems (e.g., human), comparing chromosome-wide values of mean mapping quality and depth represents a quick and often sufficient way to identify the sex chromosome complement (e.g., XX or XY) of individuals across a population. Second, in uncharacterized systems or *de novo* reference genomes, the CHROM_STATS output provides information that can help with the identification of sex-linked scaffolds. It is important to note, however, that results for both cases will vary on the basis of ploidy and with differences in the degree of sequence homology between the sex chromosomes.

The ANALYZE_BAM module runs a series of analyses designed to aid in the identification of sex-linked sequence and characterize the sex chromosome content of an individual. In doing so, it provides more detailed metrics than CHROM_STATS. For ANALYZE_BAM, XYalign runs Platypus [32] across multiple threads, if permitted, to identify variants. It then parses the output VCF file containing the variants, applies filters for site quality, genotype quality, and read depth, and plots the read balance at variant sites. Here, we define read balance at a given site as the number of reads containing the alternate allele (i.e., nonreference allele) divided by the total number of reads mapped to the position. XYalign produces plots and tables for read balance per site, as well as mean read balance and variant count per genomic bin or window across a chromosome. We anticipate that these data will not only be useful for masking regions containing incorrect genotypes but will also aid in the identification of PARs as well. XYalign next traverses the BAM file, calculating mean mapping quality and an approximation of mean depth in windows across the genome. During traversal, depth is calculated as the total length of all reads (primary alignments only) mapping to a genomic window divided by the total length of the window. We have found that this heuristic approximation is very similar to calculations of exact depth, particularly as window sizes increase, and is much faster to compute across entire chromosomes. XYalign will output a table containing genomic coordinates, mean depth, and mean mapping quality for each window. It will then filter windows on the basis of user-defined thresholds of mean depth and mapping quality and output 2 BED files containing windows that passed and failed these thresholds, respectively, which can be used for additional masking in downstream applications. Finally, XYalign will output plots of mapping quality and depth in each window across each chromosome.

After ANALYZE_BAM is run, the windows meeting thresholds can be used by the CHARACTERIZE_SEX_CHROMS module to systematically compare mean depth in pairs of chromosomes using 3 different approaches. The first is a bootstrap analysis that provides 95% confidence intervals of mean window depth for each of the chromosomes in a given pair to test for overlap. The second is a permutation analysis to test for differences in depth between the 2 chromosomes. The third is a 2-sample Kolmogorov-Smirnov test [35]. Although all 3 tests are implemented in XYalign, we only present results from the bootstrap analyses in this article. Furthermore, while we present analyses pairing sex chromosomes with an autosome (here we use chromosome 19), the chromosome pairs are arbitrary and can feature any scaffolds or chromosomes in a reference genome, depending on a user's needs.

Finally, the REMAPPING module will infer the presence or absence of a Y chromosome on the basis of the results of CHARACTERIZE_SEX_CHROMS. If a Y chromosome is not detected, the STRIP_READS module will iteratively remove reads from the sex chromosomes by read group ID using SAMtools [34], writing FASTQ files for each. XYalign will use repair.sh from BBTools to sort and re-pair paired-end reads or shuffle.sh from BBTools [30] to sort single-end reads for each read group. The REMAPPING module then maps reads with BWA-MEM [31] and sorts alignments with SAMtools [34] by read group. If >1 read group is present, the resulting BAM files are merged using SAMtools [34]. Finally, XYalign uses Sambamba [33] to isolate all scaffolds not associated with sex chromosomes from the original BAM file and then SAMtools [34] to merge this file with the BAM file containing the new sex chromosome mappings.

### Full pipeline

When run as a full pipeline on a sample, XYalign will first call PREPARE_REFERENCE to generate XX and XY reference genomes with appropriate masks. Next, it will call ANALYZE_BAM and CHARACTERIZE_SEX_CHROMS to preliminarily analyze the unprocessed input BAM file. Then, based on the results of CHARACTERIZE_SEX_CHROMS, XYalign will call STRIP_READS to extract reads from the sex chromosomes and REMAPPING to remap to the appropriate reference genome output from PREPARE_REFERENCE. Finally, XYalign will rerun the ANALYZE_BAM module to analyze the remapped BAM file and provide metrics to allow a before-and-after comparison.

While we anticipate that this full pipeline will be useful in certain situations, it is neither the only nor the best-suited option for most users. Rather, we expect that most users will call modules individually. We provide recommendations for incorporating XYalign into bioinformatic pipelines in the discussion.

### Operation

XYalign is available via PyPI [36], Bioconda [37], and Github [38], with documentation hosted at Read the Docs [39]. A full environment containing all dependencies can be most easily installed and managed using Anaconda [40] and Bioconda [37]. It has been tested on Linux and MacOS, but it is not currently supported for the Windows operating system. XYalign is typically invoked from the command line, but, as a Python library, its modules can be imported into Python scripts for more customized use cases.

## Methods

### Data

To explore the effects of sequence homology on genomic data and highlight some features of XYalign, we used 2 datasets from publicly available sources (Supplemental Table S1): (1) exome, low-coverage whole-genome, and high-coverage whole-genome sequencing data from 1 male (HG00512) and 1 female (HG00513) from the 1000 Genomes Project (Dataset 1; [41]); and (2) 24 high-coverage whole genomes from the 1000 Genomes Project (Dataset 2; [42]). For Dataset 1, we mapped reads to the hg19 version of the human reference genome [43] using BWA MEM [31], marked duplicates with SAMBLASTER [44], and used SAMtools [34] to sort, index, and merge BAM files. The publicly available BAM files for Dataset 2 were previously mapped using a different version of hg19 (from the Broad Institute's GATK Resource Bundle [45]), which we used for analyses involving this dataset.

We used the high-coverage whole-genome sequencing data from Dataset 1 to identify and understand the effects of sex chromosome homology on genomic data and analyses. We used the full Dataset 1 to observe whether patterns of depth and mapping quality can be used to identify genetic sex in a similar way across sequencing strategies (exome, low-coverage whole-genome, and high-coverage whole genome). Finally, we used Dataset 2 to test whether population data can be easily used to identify the genetic sex of individuals.

### Identifying effects of sex chromosome homology

To discover technical artifacts arising from sequence homology on the sex chromosomes and test the effects of possible corrections, we ran the full XYalign pipeline (described in Software Description) on all 6 BAM files from Dataset 1 (Supplementary Methods). We first used the PREPARE_REFERENCE module to prepare separate XX and XY versions of the hg19 reference. We then used these reference versions as input when running the full pipeline on all 6 files. In addition to masking the entire Y chromosome in the XX assembly, we also masked PAR1 and PAR2 on the Y chromosome in the XY assembly.

We explored variation in mapping quality and depth in association with genomic features on the X and Y chromosomes. On the Y chromosome, we used coordinates from Poznik et al. [46] based on Skaletsky et al. [47] (provided by D. Poznik, personal communication). On the X chromosome, we obtained coordinates for ampliconic regions from Cotter et al. [48] and all other regions (PARs, telomeres, centromere, and X-transposed region [XTR]) from the University of California Santa Cruz (UCSC) Table Browser [49]. We define the XTR on the X chromosome as beginning at the start of DXS1217 and ending at the end of DXS3 [50].

To count variants falling in major genomic regions, we first filtered VCF files with and without sex-specific mapping for each sample in Dataset 1 generated as part of the XYalign pipeline. We used BCFtools [34] to remove variants with MQ or QUAL scores <30. We then used BEDTools [26] to identify and count variants unique to each genomic region and file (Supplementary Methods; Supplemental Table S2).

### Inferring genetic sex

The successful use of sex-specific reference genomes (e.g., XX vs XY) requires accurately identifying the sex chromosome complement of a given sample. We tested 2 methods for sex chromosome identification implemented in XYalign on Datasets 1 and 2 (Supplementary Methods). First, we ran the CHARACTERIZE_SEX_CHROMS module to get detailed statistics across the length of the sex chromosomes, as well as to produce read balance histograms. We then used CHROM_STATS to test whether summary measures for each chromosome could also result in accurate assessments.

### Specific commands

We provide templates for all of the analyses described above in the Supplementary Methods. We further provide links to data and Snakemake [51] workflows for all assembly and analysis steps on Github [38] and Zenodo [52].

## Results and Discussion

### Sequence homology affects read mapping and variant calling

**Figure 1:**
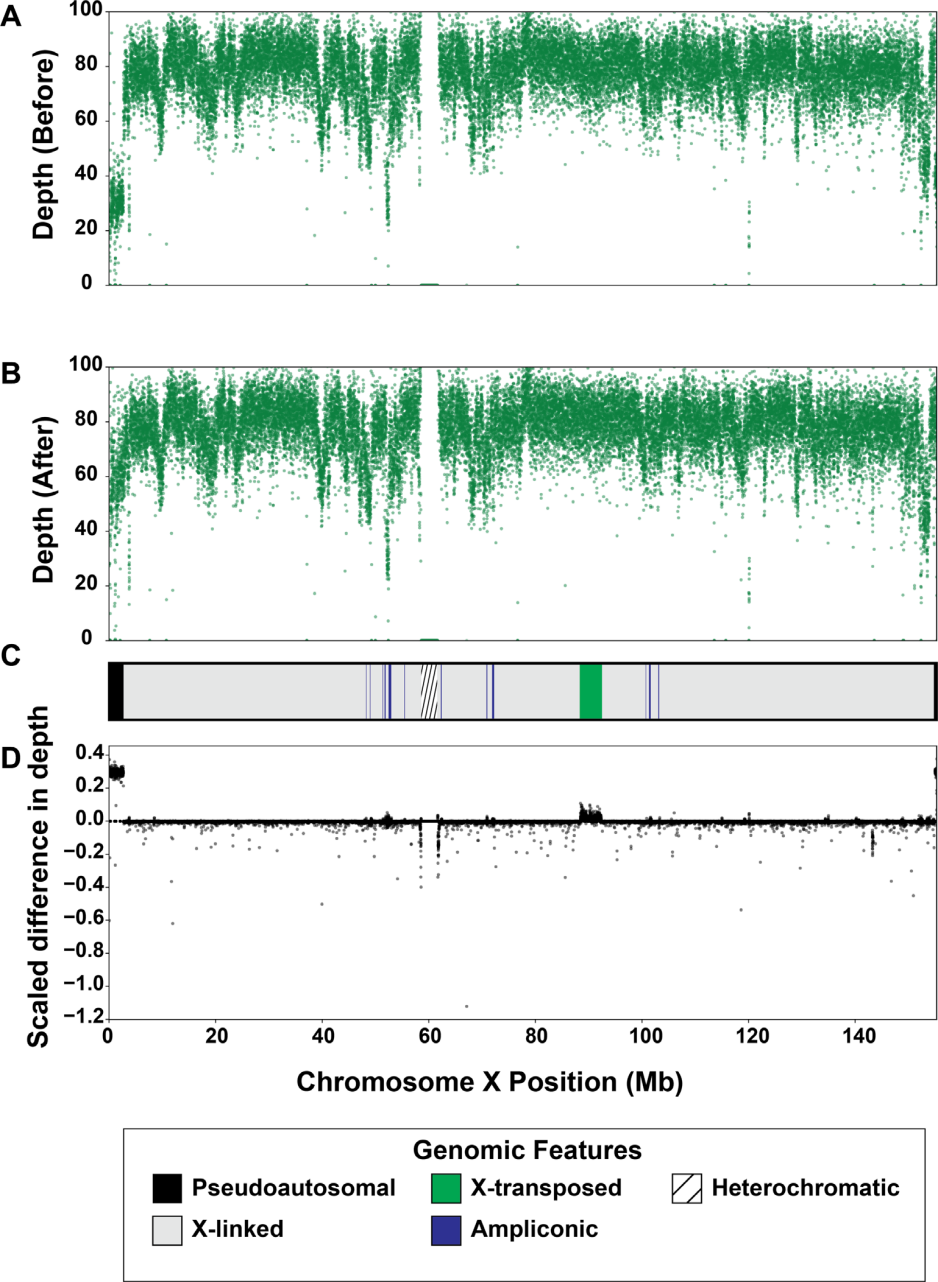
Sequencing depth on chromosome X before and after XYalign. Mean sequencing depth for the Dataset 1 XX individual in 5-kb windows across the X chromosome before (A) and after (B) XYalign processing. Changes in depth (D) are presented as the sign of the difference times the absolute value of the log_10_ difference, where the difference is depth after XYalign minus depth before XYalign. The chromosome map (C) presents the location of X chromosome genomic features depicted in the legend. X chromosome coordinates are identical in all plots.

**Figure 2:**
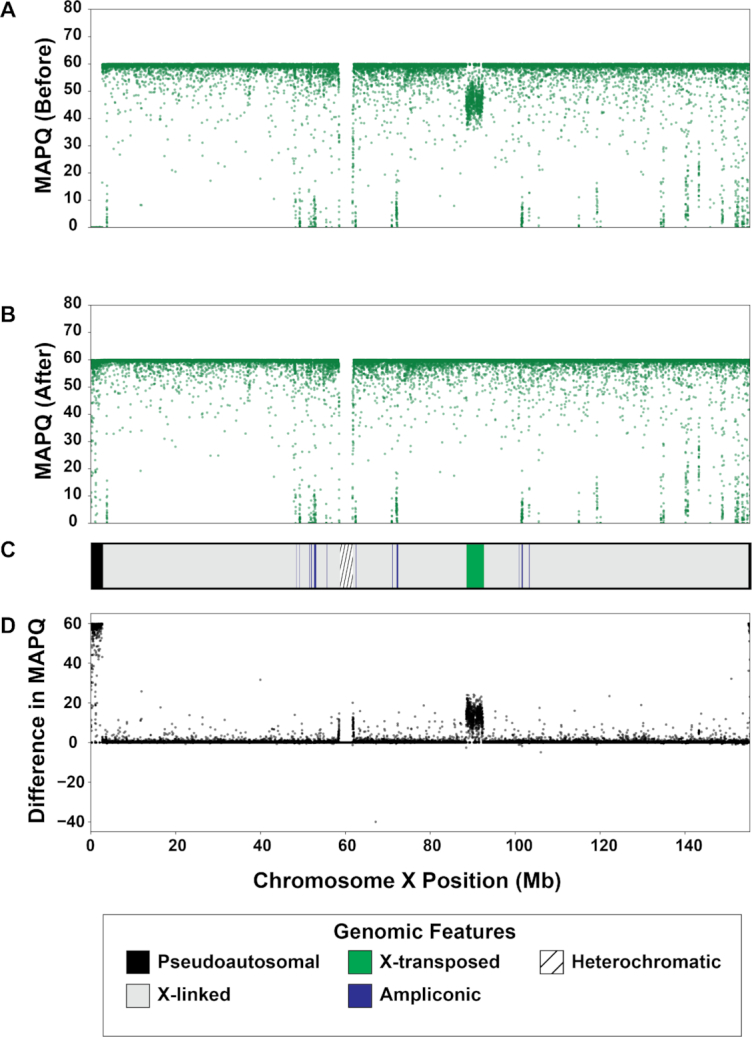
Mapping quality on chromosome X before and after XYalign. Mean mapping quality (MAPQ) for the Dataset 1 XX individual in 5-kb windows across the X chromosome before (A) and after (B) XYalign processing. Changes in MAPQ (D) are presented as the difference in MAPQ after XYalign minus MAPQ before XYalign. The chromosome map (C) presents the location of X chromosome genomic features depicted in the legend. X chromosome coordinates are identical in all plots.

**Figure 3:**
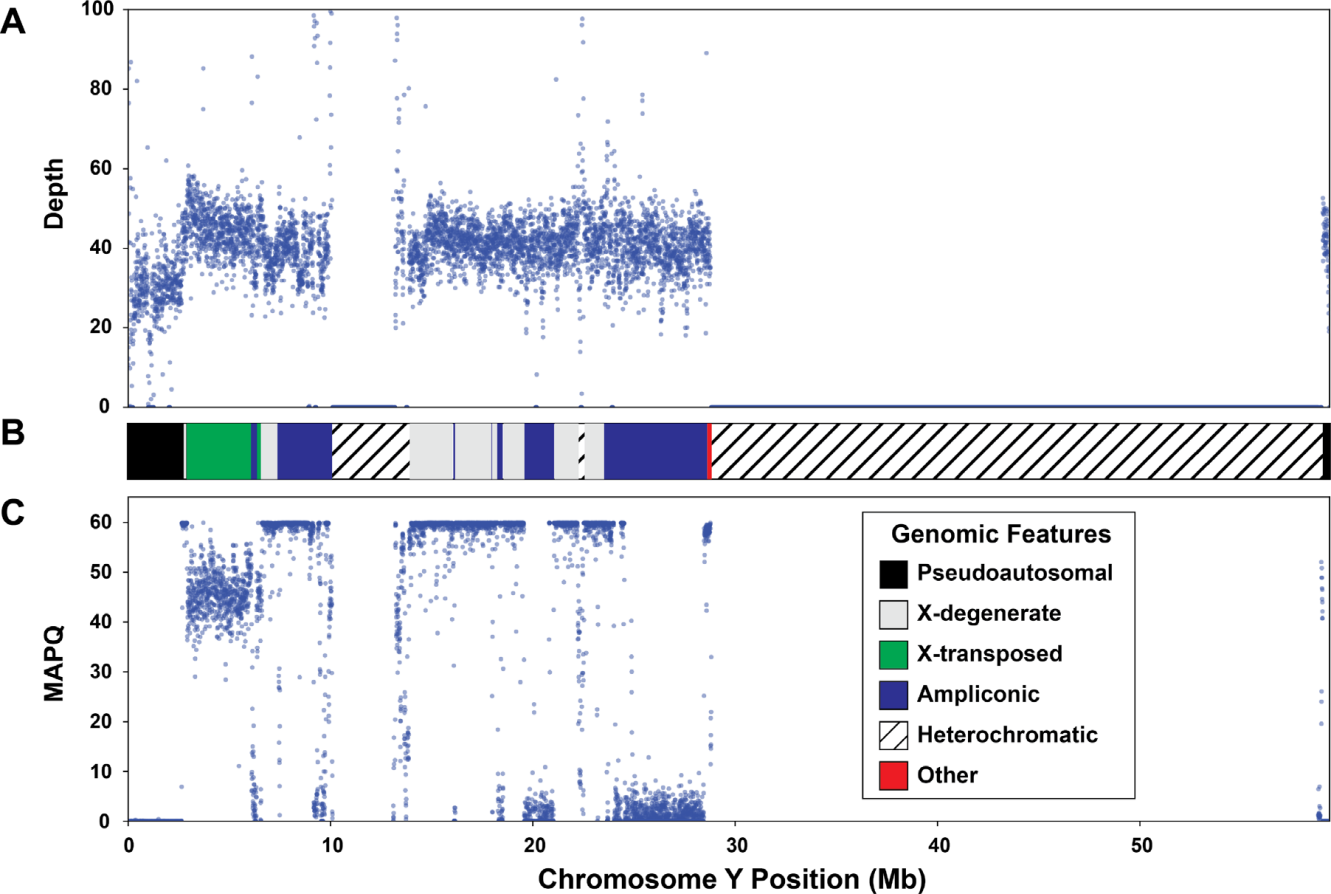
Y chromosome sequencing depth and quality. Mean sequencing depth (A) and mapping quality (MAPQ; C) for the Dataset 1 XY individual in 5-kb windows across the Y chromosome. The chromosome map (B) presents the location of Y chromosome genomic features depicted in the legend. Y chromosome coordinates are identical in all plots.

We found that sex chromosome sequence homology leaves a variety of detectable signals in the genome. First, PAR1 and PAR2 on both sex chromosomes are clearly identifiable in genomic scatter plots of mapping quality and depth in all datasets (Figs 1–3). While these results are not surprising given the sequence homology in these regions [11], they highlight the fact that these measures can help identify other similarly problematic areas. For example, there is a region of reduced mapping quality on the X chromosome beginning near 88.4 Mb and ending near 92.3 Mb (Fig. 2). This corresponds to the XTR, which arose by a duplication from the X to the Y chromosome in the human lineage since its divergence with the chimpanzee-bonobo lineage [11, 53]. This region retains >98% sequence similarity between the X and Y chromosome [11], likely leading to the reduction in mapping quality. Interestingly, we observe a similar decrease in mapping quality on the Y chromosome beginning near 2.9 Mb and ending near 6.6 Mb, corresponding with known coordinates of the XTR on the Y chromosome (Fig. 3). In fact, integrating mapping quality and depth recapitulates established genomic features of both sex chromosomes (e.g., ampliconic regions, PARs, and XTRs) described in previous studies (Figs 1–3; [46, 54]). This suggests that, in at least some cases, the output of XYalign can be used to quickly explore broad patterns of genomic architecture and mask regions likely to introduce technical difficulties in genomic analyses.

By hard-masking the Y chromosome in the XX reference genome, and the PARs (PAR1 and PAR2) in the XY reference genome, we observed clear improvements in read mapping for the XX individual (Figs 1 and 2). On the X chromosome, all metrics exhibited striking improvements in PAR1, PAR2, and XTR (Figs 1 and 2). Furthermore, the XX individual no longer had any variant calls or mapped reads on the Y chromosome, although many passed filters before XYalign processing (variants before: 4,266; variants after: 0; mapped reads before: 5,729,007; mapped reads after: 0). While this is expected given the hard masking of the Y chromosome, it is worth emphasizing that this is consistent with the biological state of the individual.

We found that these improvements in mapping on the X chromosome after masking the Y chromosome substantially affected downstream variant calling (Table 1). Unsurprisingly, the effect was most pronounced in the PARs, in which thousands of variants were callable after masking the identical sequences present on the Y chromosome in the reference assembly. The XTR also had a large increase in the number of variants detected after Y masking—a mean of 85.4 variants per megabase of sequence (Table 1). However, effects were not limited to these regions of well- documented homology: both the X-added region (XAR) and X-conserved region (XCR) contained hundreds of affected variants, suggesting effects of more extensive homology across the sex chromosomes.

**Table 1:** The effect of sex chromosome homology on variant calling on the X chromosome^a^

Region^b^	Length (bp)^c^	Before Only (per Mb)^d^	After Only (per Mb)^e^
PAR1	2,589,520	0 (0)	7,563 (2,920.6)
PAR2	329,516	0 (0)	633 (1921)
XTR	4,287,237	40 (9.3)	366 (85.4)
XAR	55,982,492	299 (5.3)	400 (7.2)
XCR	89,011,795	610 (6.9)	523 (5.9)
Total	152,250,560	949 (6.2)	9,485 (62.3)

^a^High-coverage whole-genome data from XX individual in Dataset 1.

^b^PAR1: pseudoautosomal region 1; PAR2: pseudoautosomal region 2; XTR: X-transposed region; XAR: X-added region; XCR: X-conserved region.

^c^Total sequence length of region in base pairs.

^d^Total number of variants, after filtering, present before but not after Y chromosome masking. Variants per Mb of sequence are presented in parentheses.

^e^Total number of variants, after filtering, present after but not before Y chromosome masking. Variants per Mb of sequence are presented in parentheses.

### Inferring genetic sex

**Figure 4:**
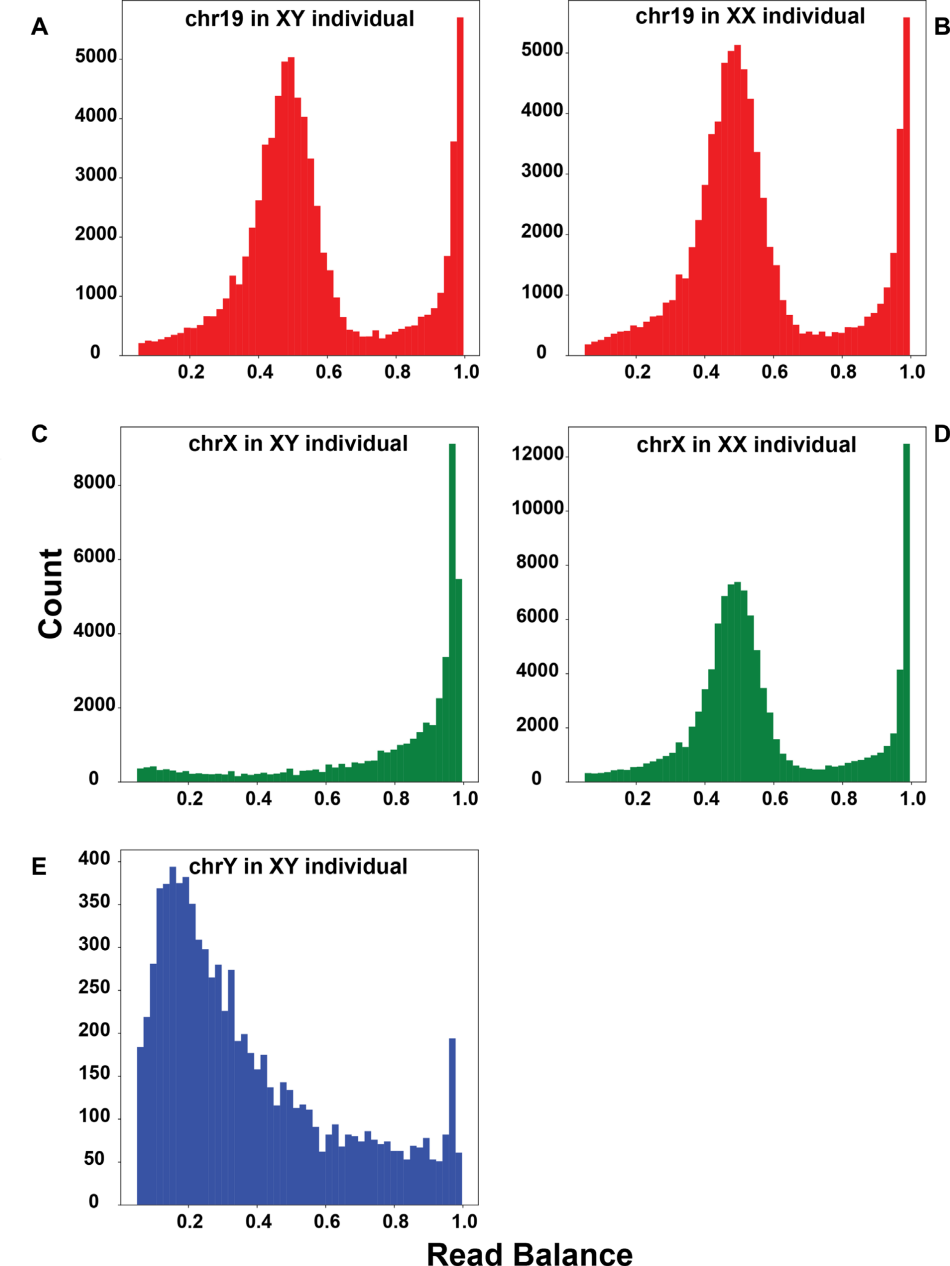
Read balance in XY and XX samples. Histograms of read balance for an XY sample (left column; A, C, and E) and XX sample (right column; B and D) from Dataset 1 across chromosome 19 (A and B), chromosome X (C and D), and chromosome Y (E). Read balance at a given site is defined as the number of reads containing a non-reference allele divided by the total number of reads mapped to a site. Read balances between 0.05 and 1.0, non-inclusive, are presented to highlight “heterozygous” read balances. Full distributions, including fixed sites, are presented in Supplemental Fig. 1.

In our analyses, the most striking measure for assessing an individual's sex chromosome complement was the distribution of read balances across a chromosome (Fig. 4). Specifically, when we plotted the distribution of the fraction of reads containing a nonreference allele at a given variant site, we observed that diploid chromosomes (e.g., autosomes, and chromosome X in XX individuals) exhibited peaks around both 0.5 and 1.0, consistent with the presence of heterozygous sites and sites homozygous for a nonreference allele, respectively (Fig. 4). In the case of the X chromosome in XY individuals, we observed a single peak near 1.0, consistent with an expected haploid state (i.e., no heterozygous sites; Fig. 4). We observed 1 exception to this pattern: the Y chromosome exhibited a peak around 0.2 in addition to the one near 1.0 (Fig. 4; Supplemental Fig. 1). All variants included in analyses met thresholds for depth, site quality, and genotype quality, so quality does not seem to be a driving factor of this pattern. This pattern also remained after genomic windows of low mapping quality and irregular depth were removed. When we parsed variants by Y chromosome region, we discovered that this pattern appears in ampliconic, heterochromatic, and XTR regions, while X-degenerate regions display our expected haploid expectation of a single peak close to 1.0 (Supplemental Figs S2−S5). Moreover, there are fewer sites in the X-degenerate regions than the other bins (Supplemental Table S3). While, taken together, this explains the peak around 0.2 when looking across the entire chromosome (Fig. 4; Supplemental Fig. 1), we are currently unable to explain the specific factors causing the peak near 0.2 in these regions. Homology is likely playing a role because both the ampliconic and heterochromatic regions are highly repetitive and the XTR shares homology with the X chromosome. However, more work is required to explore this possibility in more detail and, further, to understand how homology can cause this pattern and lead to what seem to be false-positive variants passing all filters. It will additionally be important to determine whether similar results are obtained on the W chromosome in ZW systems.

**Figure 5:**
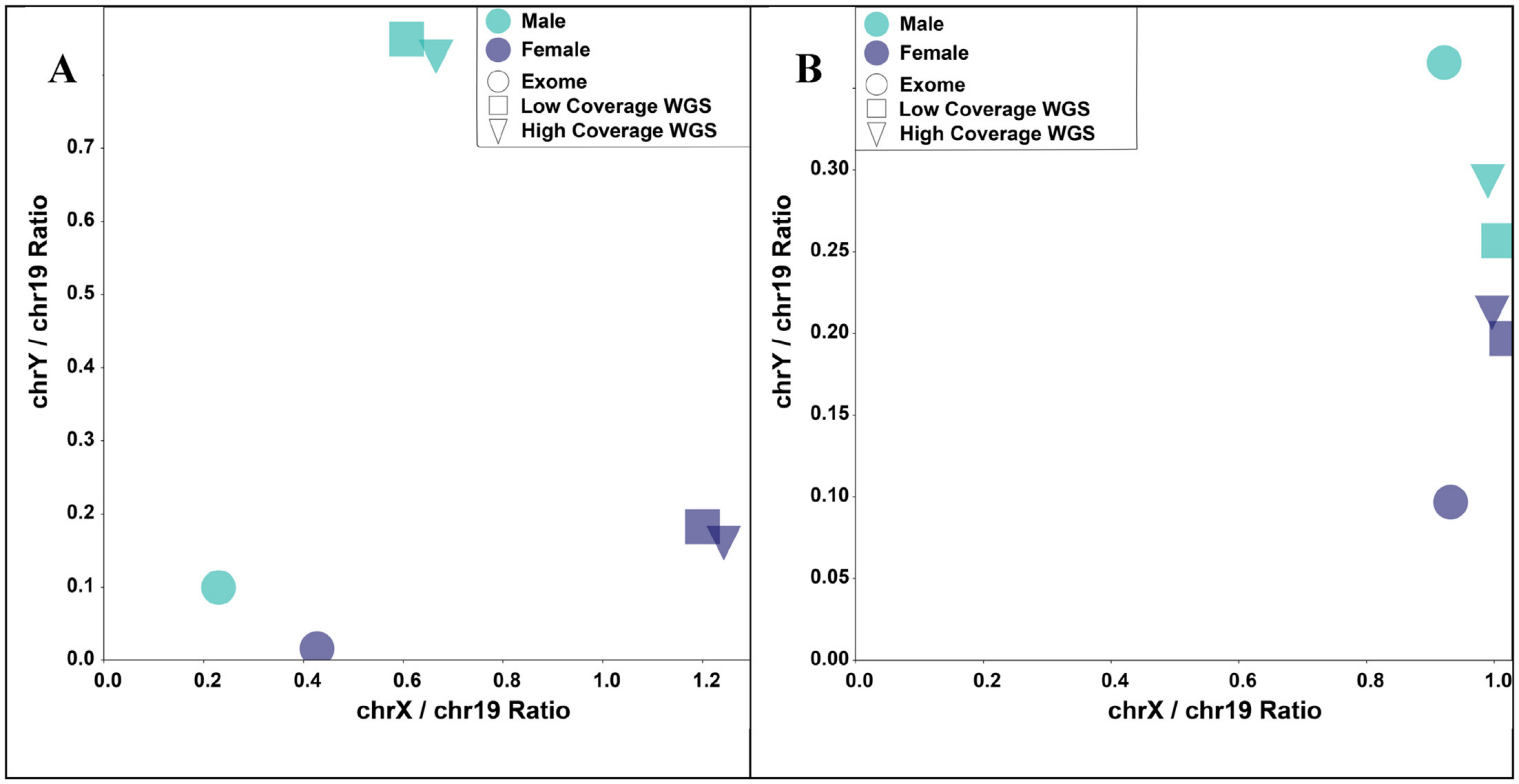
Relative sequencing depth and mapping quality on the X and Y chromosomes across different sequencing strategies. Values of relative (A) sequencing depth and (B) mapping quality come from exome (circles), low-coverage whole-genome sequencing (WGS; squares), and high-coverage WGS (triangles) for a single male (green) and female (blue) individual. Mean depth and MAPQ on chromosome 19 was used to normalize the sex chromosomes.

Across datasets, we observed variation in relative depth of the X and Y chromosomes in XX and XY individuals, particularly among different sequencing strategies: exome, low-coverage whole-genome, and high-coverage whole-genome sequencing (Fig. 5A). However, within datasets, XX and XY individuals were clearly differentiated (Fig. 5; Supplemental Fig. 6). This pattern suggests that a general threshold for assigning different genetic sexes across a range of organisms and sequencing experiments might be difficult to implement. That being said, within species, some combination of depth, mapping quality, and read balance is likely to be informative. For example, in humans, relative mapping quality appears to be informative in some sequencing strategies, particularly exome sequencing (Fig. 5B). However, this should be explored in each experiment, as we did not observe this differentiation in the uncorrected 1000 Genomes high-coverage samples (Supplemental Fig. S7).

Generating these results for all individuals in a study is easy to do with XYalign: one can iteratively run the CHARACTERIZE_SEX_CHROMS module on preliminarily mapped BAM files. Then, the results from all individuals can be analyzed together. At least with human samples, for which X and Y chromosomes are very differentiated, this process can be sped up significantly with the CHROM_STATS module. In our data, read counts on the X and Y chromosomes quickly and clearly clustered male and female samples within sequencing strategies (i.e., exome, low-coverage whole-genome, and high-coverage whole-genome; Supplemental Figs S8 and S9). However, the success of this procedure likely depends on the degree of differentiation between sex chromosomes; other organisms might require the statistics output as part of the CHARACTERIZE_SEX_CHROMS module.

### Recommendations for researchers

Based on these results, we can make the following recommendations for researchers. For organisms with multiple sex chromosomes assembled (e.g., both X and Y or both Z and W) and included in reference assemblies (e.g., human, chimpanzee, rhesus macaque, gorilla, mouse, rat, chicken, *Drosophila*), if the genetic sex of every individual is known, the user may (1) prepare separate assemblies for the different sexes using the PREPARE_REFERENCE module, (2) map and process reads according to the user's typical pipeline (mapping individuals by sex to their corresponding reference), (3) confirm genetic sex using the CHROM_STATS module, (4) remap any incorrectly assigned individuals, and (5) proceed with downstream analyses. If genetic sexes of individuals are unknown, the user should then (1) prepare separate assemblies for the different sexes using the PREPARE_REFERENCE module; (2) map and process a suitable number of reads (e.g., whole dataset for exome or a single lane of WGS) according to the user's typical pipeline using the reference genome of the heterogametic sex (i.e., XY or ZW); (3) infer the sex chromosome complement using either CHROM_STATS (for well-characterized and highly divergent sex chromosomes), CHARACTERIZE_SEX_CHROMS, or both; (4) map and process all reads using the prepared reference genome corresponding to the inferred sex of each individual; and (5) run downstream analyses.

For individuals of the homogametic sex (i.e., XX or ZZ), the above recommendations will likely completely remove artifacts stemming from sex chromosome homology, assuming only a single unmasked sex chromosome is left after XYalign processing. However, homology is unavoidable for individuals of the heterogametic sex (i.e., XY or ZW) because both sex chromosomes are required in the reference assembly for mapping. In this case, a more local masking or filtering approach is likely the most promising option. For studies investigating specific variants, for which false-negative results are preferable to false-positive results, we suggest strict variant filtering that includes high thresholds for mapping quality (e.g., thresholds of ≥55 are required to eliminate the effects of homology in the XTR). However, for studies investigating invariant sites as well (e.g., measures of genetic diversity require information from all monomorphic and polymorphic sites), we recommend filtering entire regions based on, at the very least, mapping and depth metrics. These masks are output by the BAM_ANALYSIS module in XYalign, and for this use, we recommend using small windows (e.g, 1−5 kb) and exploring a variety of depths. Finally, in all cases, if PARs are present in the reference genome, they should be masked in the heterogametic sex's assembly output by the PREPARE_REFERENCE module.

### Additional uses for XYalign

While the development of XYalign was motivated by challenges surrounding erroneous read mapping and variant calling due to sex chromosome homology in human sequencing experiments, the software can be utilized in a number of additional scenarios. First, it can be applied to any species with heteromorphic sex chromosomes to identify relative quality and depth. The results output by CHROM_STATS, ANALYZE_BAM, and CHARACTERIZE_SEX_CHROMS can be used to identify sex-linked scaffolds, characterize sex chromosome complements, and determine the most appropriate remapping strategy. Second, XYalign can be used to detect relative sequencing depth, mapping quality, and read balance on any chromosome, not just the sex chromosomes. In addition to exploring mapping artifacts, we anticipate that this will aid in the detection of aneuploidy in the autosomes. However, we note that many programs exist to calculate depth of coverage (e.g., [26, 55, 56]) and identify structural variants within statistical frameworks (e.g., [57–60]). Accordingly, XYalign might not be the most appropriate option for detecting local phenomena such as copy number variants. Finally, XYalign may also be extended to other types of data, including RNA sequencing data, where the same fundamental challenge (gametologous sequence between the X and Y) can affect mapping and variant calling. In particular, we expect artifacts to manifest in differential expression and biased-allelic expression, and suggest that the PREPARE_REFERENCE module be considered for all RNA sequencing experiments in systems with sex chromosomes.

## Conclusion

We showed that the complex evolutionary history of the sex chromosomes creates mapping artifacts in next-generation sequencing data that have downstream effects on variant calling and other analyses. These technical artifacts are likely present in most genomic datasets of species with chromosomal sex determination and may be pervasively affecting genomic analyses on the sex chromosomes. However, many of these artifacts can be corrected through the strategic use of masks during read mapping and the filtering of variants. We developed XYalign, a tool that facilitates the characterization of an individual's sex chromosome complement and implements this masking strategy to correct these technical artifacts. We illustrated how XYalign can be used to identify the presence or absence of a Y chromosome, characterize biases in mapping across the genome, and correct for these mapping artifacts. XYalign provides a reproducible framework to generate more robust short-read mapping and improve variant calling on the sex chromosomes.

## Availability of supporting data and materials

XYalign is available on Github [38] under a GNU General Public License (version 3). We have also deposited a static version of the source code used for analyses in this paper at Zenodo [52].

## Availability of supporting source code and requirements

Project name: XYalign

Project home page: https://github.com/SexChrLab/XYalign

Operating systems: Linux and MacOS

Programming Language: Python

Other requirements: Matplotlib, NumPy, Pandas, PyBedTools, PySam, SciPy, BBTools, BWA, Platypus, Sambamba, and SAMtools

License: GNU GPL v3

RRID:SCR_016661

## Additional files

Supplementary Methods.

Table S1. Samples included in this study.

Table S2. Coordinates of major X chromosome features in hg19.

Table S3. Variants identified across the Y chromosome.

Figure S1. Read balance in XY and XX samples including fixed sites.

Figure S2. Read balance in ampliconic regions of the Y chromosome.

Figure S3. Read balance in heterochromatic regions of the Y chromosome.

Figure S4. Read balance in X degenerate regions of the Y chromosome.

Figure S5. Read balance in X-transposed regions of the Y chromosome.

Figure S6. Relative sequencing depth on the X and Y chromosomes in the 1000 Genomes Project high-coverage samples.

Figure S7. Relative mapping quality (MAPQ) on the X and Y chromosomes in the 1000 Genomes Project high-coverage samples.

Figure S8. Relative number of reads mapped to the X and Y chromosomes across different sequencing strategies.

Figure S9. Relative number of reads mapped to the X and Y chromosomes in the 1000 Genomes Project high-coverage samples.

giz074_GIGA-D-18-00312_Original_Submission

giz074_GIGA-D-18-00312_Revision_1

giz074_GIGA-D-18-00312_Revision_2

giz074_GIGA-D-18-00312_Revision_3

giz074_Response_to_Reviewer_Comments_Original_Submission

giz074_Response_to_Reviewer_Comments_Revision_1

giz074_Response_to_Reviewer_Comments_Revision_2

giz074_Reviewer_1_Report_Original_Submission Qi Zhou -- 9/1/2018 Reviewed

giz074_Reviewer_1_Report_Revision_1 Qi Zhou -- 12/18/2018 Reviewed

giz074_Reviewer_2_Report_Original_Submission Kristoffer Sahlin -- 9/3/2018 Reviewed

giz074_Reviewer_2_Report_Revision_1 Kristoffer Sahlin -- 12/13/2018 Reviewed

giz074_Supplemental_Files

## Abbreviations

BAM: Binary Alignment Map; bp: base pairs; BWA: Burrows-Wheeler Aligner; GATK: Genome Analysis Toolkit; kb: kilobase pairs; MAPQ: mean mapping quality; Mb: megabase pairs; Pandas: Python Data Analysis Library; PAR: pseudoautosomal region; PyPI: Python Package Index; UCSC: University of California Santa Cruz; VCF: Variant Call Format; WGS: whole-genome sequencing; XAR: X-added region; XCR: X-conserved region; XTR: X-transposed region.

## Competing interests

The authors declare that they have no competing interests.

## Funding

This study was supported by startup funds from the School of Life Sciences and the Biodesign Institute at Arizona State University to M.A.W. Furthermore, this study was supported by the National Institute of General Medical Sciences of the National Institutes of Health under Award No. R35GM124827 to M.A.W. The content is solely the responsibility of the authors and does not necessarily represent the official views of the National Institutes of Health.

## Authors’ contributions

M.A.W. and T.H.W. conceived the research. All authors participated in the initial design of the software. T.H.W. was responsible for subsequent design, development, and implementation of the software. B.G., E.K., T.N.P., W.W., and T.H.W. tested the software. T.H.W. analyzed the data. T.H.W. and M.A.W. wrote the manuscript. All authors were involved in the revision of the manuscript and have agreed to the final content.
