# Supplementary material for: Identifying, understanding, and correcting technical artifacts on the sex chromosomes in next-generation sequencing data
Source: Gigascience. 2019 Jul 9;8(7):giz074. doi: 10.1093/gigascience/giz074 (PMC6615978; doi:10.1093/gigascience/giz074)
Supplement: giz074_Supplemental_Files [file giz074_supplemental_files.zip › 20190705_Webster_etal_XYalign_supplement_Gigascience.docx]

**Title:**

Identifying, understanding, and correcting technical biases on the sex chromosomes in next-generation sequencing data

**Authors and Affiliations:**

Timothy H. Webster, Madeline Couse, Bruno M. Grande, Eric Karlins, Tanya N. Phung, Phillip A. Richmond, Whitney Whitford, Melissa A. Wilson

| **Supplementary Item** | **Page** |
| --- | --- |
| Supplementary Methods | 2 |
| Table S1. Samples included in this study. | 5 |
| Table S2. Coordinates of major X chromosome features in hg19. | 6 |
| Table S3. Variants identified across the Y chromosome. | 7 |
| Figure S1. Read balance in XY and XX samples including fixed sites. | 8 |
| Figure S2. Read balance in ampliconic regions of the Y chromosome. | 9 |
| Figure S3. Read balance in heterochromatic regions of the Y chromosome. | 10 |
| Figure S4. Read balance in X degenerate regions of the Y chromosome. | 11 |
| Figure S5. Read balance in X-transposed regions of the Y chromosome. | 12 |
| Figure S6. Relative sequencing depth on the X and Y chromosomes in the 1000 Genomes Project high-coverage samples. | 13 |
| Figure S7. Relative mapping quality (MAPQ) on the X and Y chromosomes in the 1000 Genomes Project high-coverage samples. | 14 |
| Figure S8. Relative number of reads mapped to the X and Y chromosomes across different sequencing strategies. | 15 |
| Figure S9. Relative number of reads mapped to the X and Y chromosomes across in the 1000 Genomes Project high-coverage samples. | 16 |

**Supplementary Methods**

Because we ran XYalign on many samples, we provide templates for commands here. In all cases, the exact commands are included in a Snakemake (Köster and Rahmann, 2012) pipeline available with the XYalign software distribution available at Github (https://github.com/WilsonSayresLab/XYalign). A permanent static version is deposited at Zenodo as well (Webster *et al.*, 2018).

*Full XYalign pipeline on Dataset 1*

We initially prepared separate XX and XY references using the following command:

xyalign --PREPARE_REFERENCE --ref <hg19 reference genome> --xx_ref_out hg19.XXonly.fasta --xy_ref_out hg19.XY.fasta --output_dir <output_directory> --x_chromosome chrX --y_chromosome chrY --bwa_index True

where *<*hg19 reference genome*>* was the path to the FASTA file containing the hg19 reference, *<*input bam file*>* was a sorted BAM file, and *<*output directory*>* was the directory where XYalign wrote output. We then ran the full pipeline on all six files from Dataset 1 using the following command template:

xyalign --ref <hg19 reference genome> --bam <input bam file> --output_dir <output directory> --sample_id <sample ID> --cpus 4 --reference_mask hg19_PAR_Ymask_startEnd.bed --window_size 5000 --chromosomes chr19 chrX chrY --x_chromosome chrX --y_chromosome chrY --xmx 4g --fastq_compression 4 --min_depth_filter 0.2 --max_depth_filter 2 --xx_ref_in hg19.XXonly.fasta --xy_ref_in ref_out hg19.XY.fasta,

where *<*sample ID*>* was the identification code for a given sample, hg19_PAR_Ymask_startEnd.bed was a BED file containing the genomic coordinates of the PARs in the hg19 assembly, and hg19.XXonly.fasta and hg19.XY.fasta were the two FASTA formatted reference genomes prepared in the previous step.

*Variant analyses on Dataset 1*

To count variants falling in major genomic regions, we intersected a BED file containing coordinates with VCF files using BEDTools (Quinlan and Hall, 2010). We first filtered VCF files using BCFtools (Li *et al.*, 2009) with the following command template:

bcftools filter --include ‘INFO/MQ>=30 && %QUAL>=30’ <input_vcf>

We then identified variants unique to each file through iterations of the “subtract” command in BEDtools (Quinlan and Hall, 2010):

bedtools subtract -header -a <first_vcf> -b <second_vcf>

Finally, in each region, we counted variants present in a given filtered VCF file using the BEDtools (Quinlan and Hall, 2010) “intersect” command:

bedtools intersect -c -a <BED file> -b <vcf_file>

where *<*BED_file*>* is the BED file containing genomic coordinates (Supplemental Table S2).

*Inferring Genetic Sex*

Next, we examined how the metrics generated by XYalign can be used to identify the sex chromosome complement of individuals from both datasets. Here, we used the CHARACTERIZE_SEX_CHROMS module of XYalign. This was automatically done for Dataset 1 when running the full pipeline (see above). For Dataset 2, we used the following command template for BAM files:

xyalign --CHARACTERIZE_SEX_CHROMS --ref <1000 genomes reference genome> --bam <input bam file> --output_dir <output directory> --sample_id <sample ID> --cpus 4 --window_size 5000 --chromosomes 19 X Y --x_chromosome X --y_chromosome Y

Finally, we explored the utility of the CHROM_STATS module for identifying sex chromosome complement and potentially sex-linked scaffolds with both datasets using the following command template for BAM files:

xyalign --CHROM_STATS --chromosomes chr1 chr8 chr19 chrX chrY chrM --bam <input_bam_1> <input_bam_2> <input_bam_3> --ref null --sample_id <name_of_analysis> --output_dir <output_dir>

We additionally ran CHROM_STATS using the above command with the addition of the “--use_counts” flag to calculate metrics using only the number of reads mapping to each chromosome.

We visualized all CHROM_STATS results using the plot_count_stats utility, with the command template:

plot_count_stats --input <chrom_stats output file> --output_prefix <output prefix>--meta <metadata text file> --exclude_suffix <suffix> --first_chr chrX --second_chr chrY --const_chr chr19 --var1_marker color --var1_marker_vals darklateblue thistle --var2_marker shape --var2_marker_vals o s v --marker_size 1700 --legend_marker_scale 0.4

where *<chrom_stats_output_file>* was either the count, mapping quality, or depth output of CHROM_STATS, *<*metadata text file*>* was the appropriate metadata text file, and *<*suffix*>* was the string to remove from filenames.

**Table S1. Samples included in this study.**

| **ID** | **Sex^a^** | **Sequencing^b^** | **Dataset^c^** | **Citation^d^** |
| --- | --- | --- | --- | --- |
| HG00512 | M | E, LC, DC | Dataset 1 | 1 |
| HG00513 | F | E, LC, DC | Dataset 1 | 1 |
| HG00419 | F | DC | Dataset 2 | 2 |
| NA20845 | M | DC | Dataset 2 | 2 |
| NA19625 | F | DC | Dataset 2 | 2 |
| NA19017 | F | DC | Dataset 2 | 2 |
| HG03052 | F | DC | Dataset 2 | 2 |
| HG01595 | F | DC | Dataset 2 | 2 |
| NA18525 | F | DC | Dataset 2 | 2 |
| NA20502 | F | DC | Dataset 2 | 2 |
| HG02568 | F | DC | Dataset 2 | 2 |
| NA18939 | F | DC | Dataset 2 | 2 |
| HG03642 | F | DC | Dataset 2 | 2 |
| HG00759 | F | DC | Dataset 2 | 2 |
| HG01112 | M | DC | Dataset 2 | 2 |
| HG01583 | M | DC | Dataset 2 | 2 |
| HG01051 | M | DC | Dataset 2 | 2 |
| HG00268 | F | DC | Dataset 2 | 2 |
| HG03742 | M | DC | Dataset 2 | 2 |
| NA19648 | F | DC | Dataset 2 | 2 |
| HG00096 | M | DC | Dataset 2 | 2 |
| HG02922 | F | DC | Dataset 2 | 2 |
| HG01565 | M | DC | Dataset 2 | 2 |
| HG01879 | M | DC | Dataset 2 | 2 |
| HG01500 | M | DC | Dataset 2 | 2 |
| HG03006 | M | DC | Dataset 2 | 2 |

^a^Reported sex.

^b^Sequencing strategy: exome (E), low-coverage whole-genome (LC), and deep-coverage whole-genome (DC).

^c^Dataset membership for this study.

^d^1. The 1000 Genomes Project Consortium, 2015. 2. Sudmant *et al.*, 2015. References in main text.

**Table S2. Coordinates of major X chromosome features in hg19.^a^**

| Chromosome | Start | End | Feature^b^ |
| --- | --- | --- | --- |
| chrX | 60000 | 2649520 | PAR1 |
| chrX | 2649520 | 58632012 | XAR |
| chrX | 61632012 | 88395830 | XCR1 |
| chrX | 88395830 | 92683067 | XTR |
| chrX | 92683067 | 154931044 | XCR2 |
| chrX | 154931044 | 155260560 | PAR2 |

^a^Coordinates in zero-index, half open format (i.e., bed format).

^b^PAR1: pseudoautosomal region 1; XAR: X-added region; XCR: X-conserved region; XTR: X-transposed region.

**Table S3. Variants identified across the Y chromosome.^a^**

| Regions^b^ | Variant sites passing filters^c^ | Mean read balance |
| --- | --- | --- |
| Ampliconic | 2606 | 0.39 |
| Heterochromatic | 4475 | 0.36 |
| Other | 168 | 0.49 |
| Pseudoautosomal | 15 | 0.82 |
| X degenerate | 1262 | 0.75 |
| X transposed | 752 | 0.57 |

^a^Variant sites (sites with a non-reference allele) identified on the Y chromosome in whole-genome sequencing data from the XY individual in Dataset 1

^b^Regions from Poznik et al. 2013

^c^Depth >= 4, GQ >= 30, QUAL >= 30

^d^Read balance at a given site is defined as the number of reads containing a non-reference allele divided by the total number of reads mapped to a site.

**
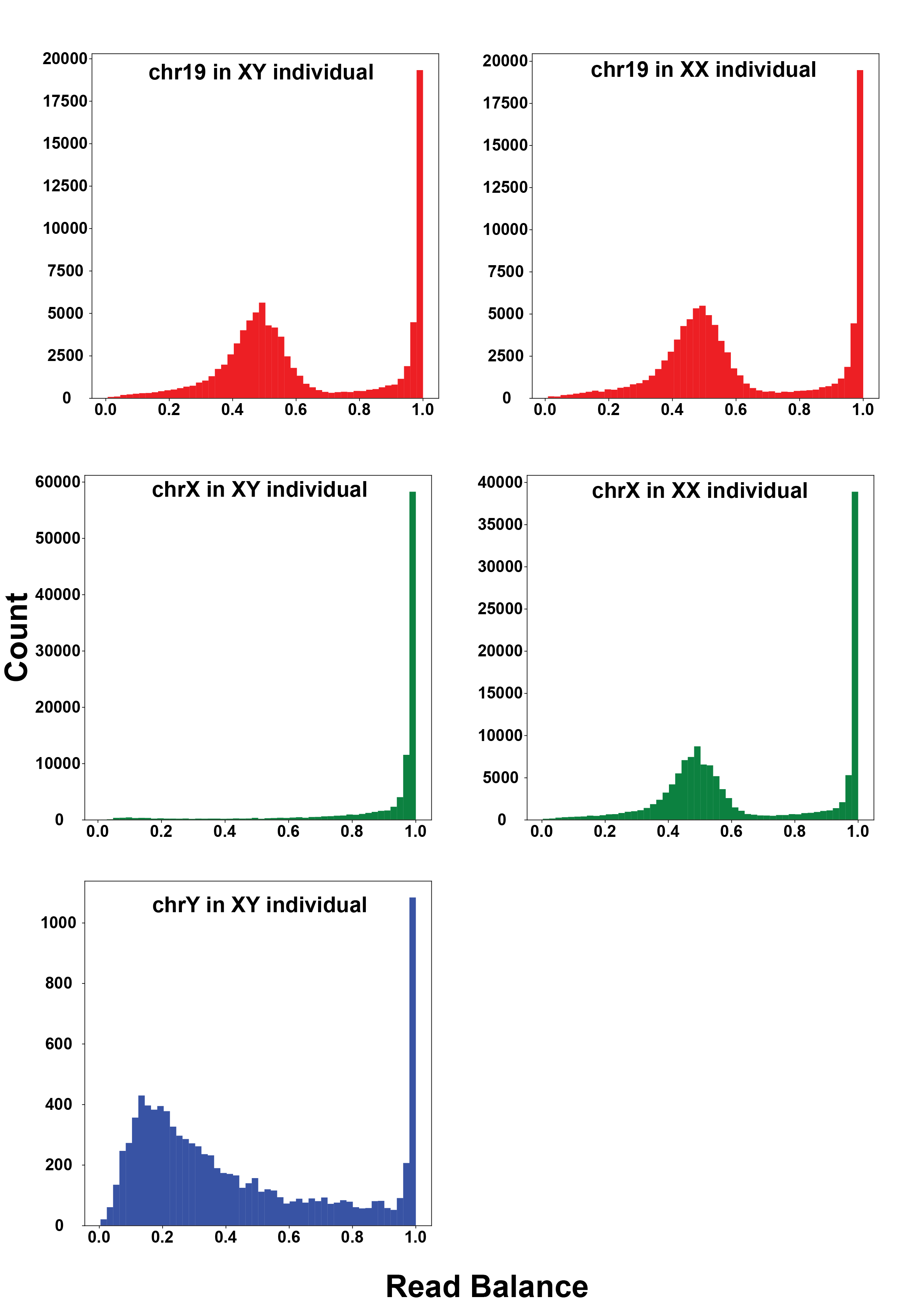
**

**Figure S1.** **Read balance in XY and XX samples including fixed sites.** Histograms of read balance for an XY sample (Left Column; A, C, and E) and XX sample (Right Column; B and D) from Dataset 1 across chromosome 19 (Top; A and B), chromosome X (Middle; C and D), and chromosome Y (Bottom; E). Read balance at a given site is defined as the number of reads containing a non-reference allele divided by the total number of reads mapped to a site. The full distribution of read balances is presented.

**Figure S2. Read balance in ampliconic regions of the Y chromosome.** Histogram of read balance for an XY sample from Dataset 1 across Y chromosome ampliconic regions. Read balance at a given site is defined as the number of reads containing a non-reference allele divided by the total number of reads mapped to a site. The full distribution of read balances is presented.

**Figure S3. Read balance in heterochromatic regions of the Y chromosome.** Histogram of read balance for an XY sample from Dataset 1 across Y chromosome heterochromatic regions. Read balance at a given site is defined as the number of reads containing a non-reference allele divided by the total number of reads mapped to a site. The full distribution of read balances is presented.

**Figure S4. Read balance in X degenerate regions of the Y chromosome.** Histogram of read balance for an XY sample from Dataset 1 across X degenerate regions of the Y chromosome. Read balance at a given site is defined as the number of reads containing a non-reference allele divided by the total number of reads mapped to a site. The full distribution of read balances is presented.

**Figure S5. Read balance in X-transposed regions of the Y chromosome.** Histogram of read balance for an XY sample from Dataset 1 across X-transposed regions of the Y chromosome. Read balance at a given site is defined as the number of reads containing a non-reference allele divided by the total number of reads mapped to a site. The full distribution of read balances is presented.


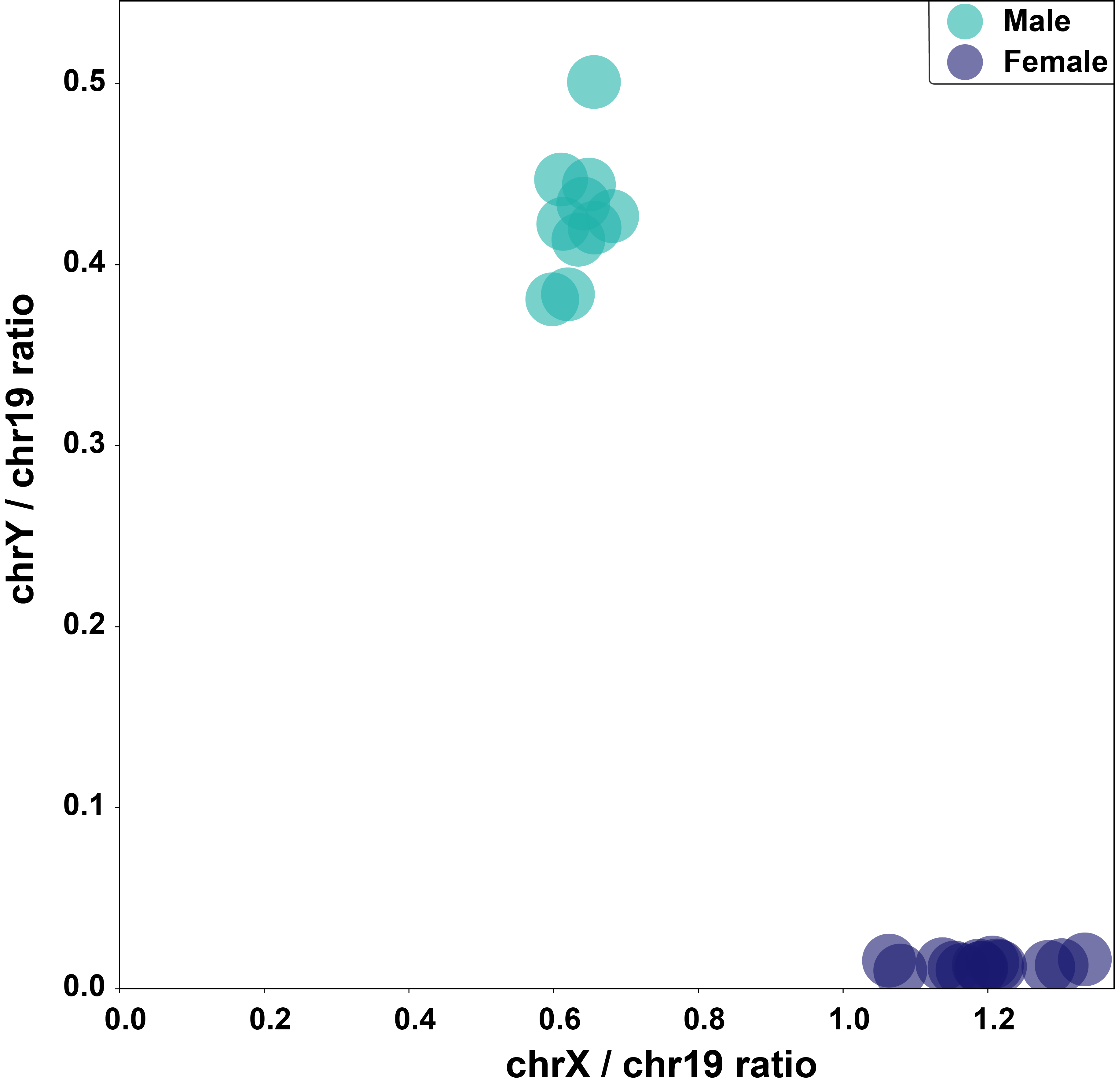


**Figure S6. Relative sequencing depth on the X and Y chromosomes in the 1000 Genomes Project high-coverage samples.** Males are plotted in green, while females are plotted in blue (Dataset 2; Table S1). Mean depth on chromosome 19 was used to normalize the sex chromosomes.


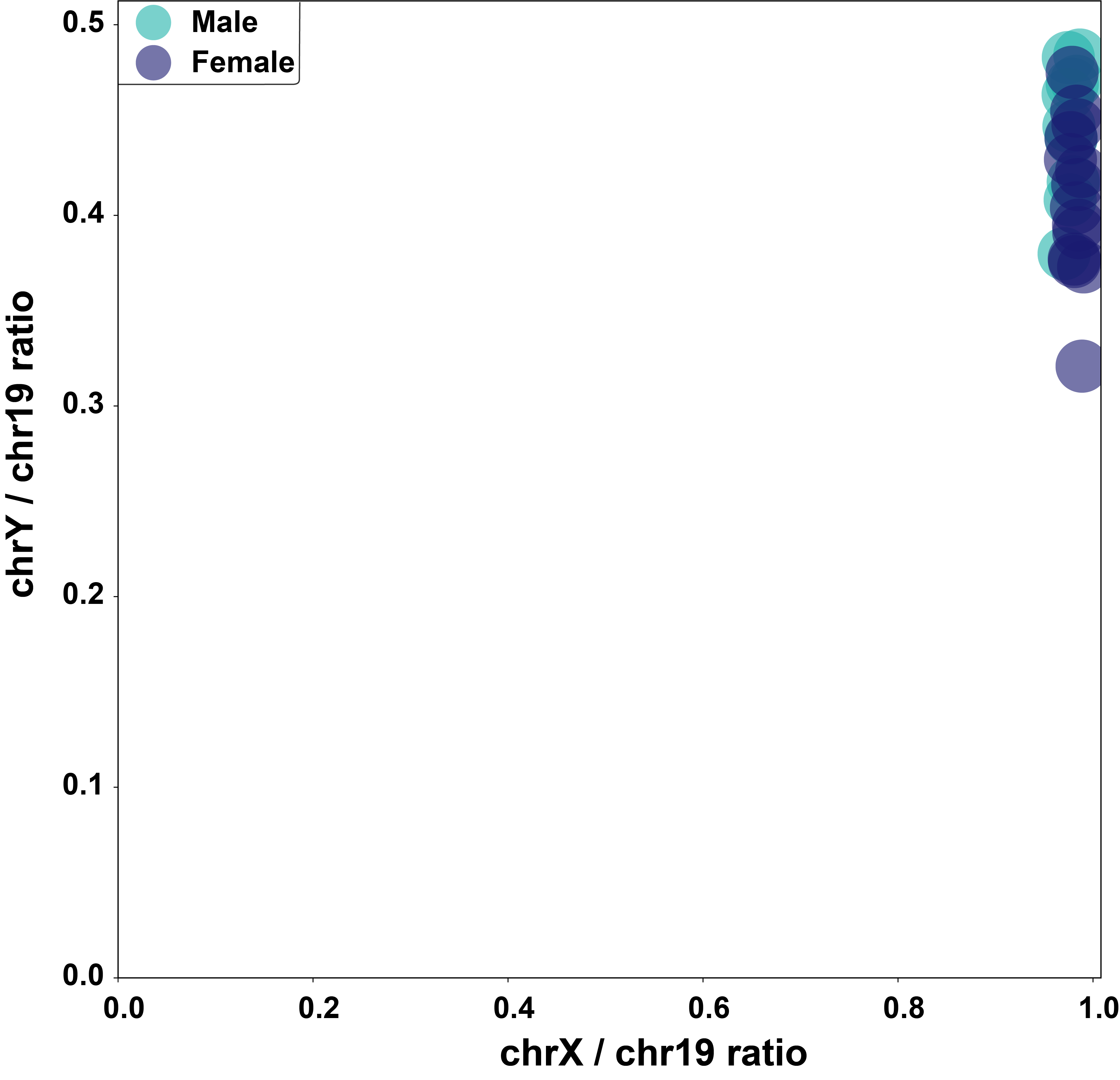


**Figure S7. Relative mapping quality (MAPQ) on the X and Y chromosomes in the 1000 Genomes Project high-coverage samples.** Males are plotted in green, while females are plotted in blue (Dataset 2; Table S1). Mean MAPQ on chromosome 19 was used to normalize the sex chromosomes.

**
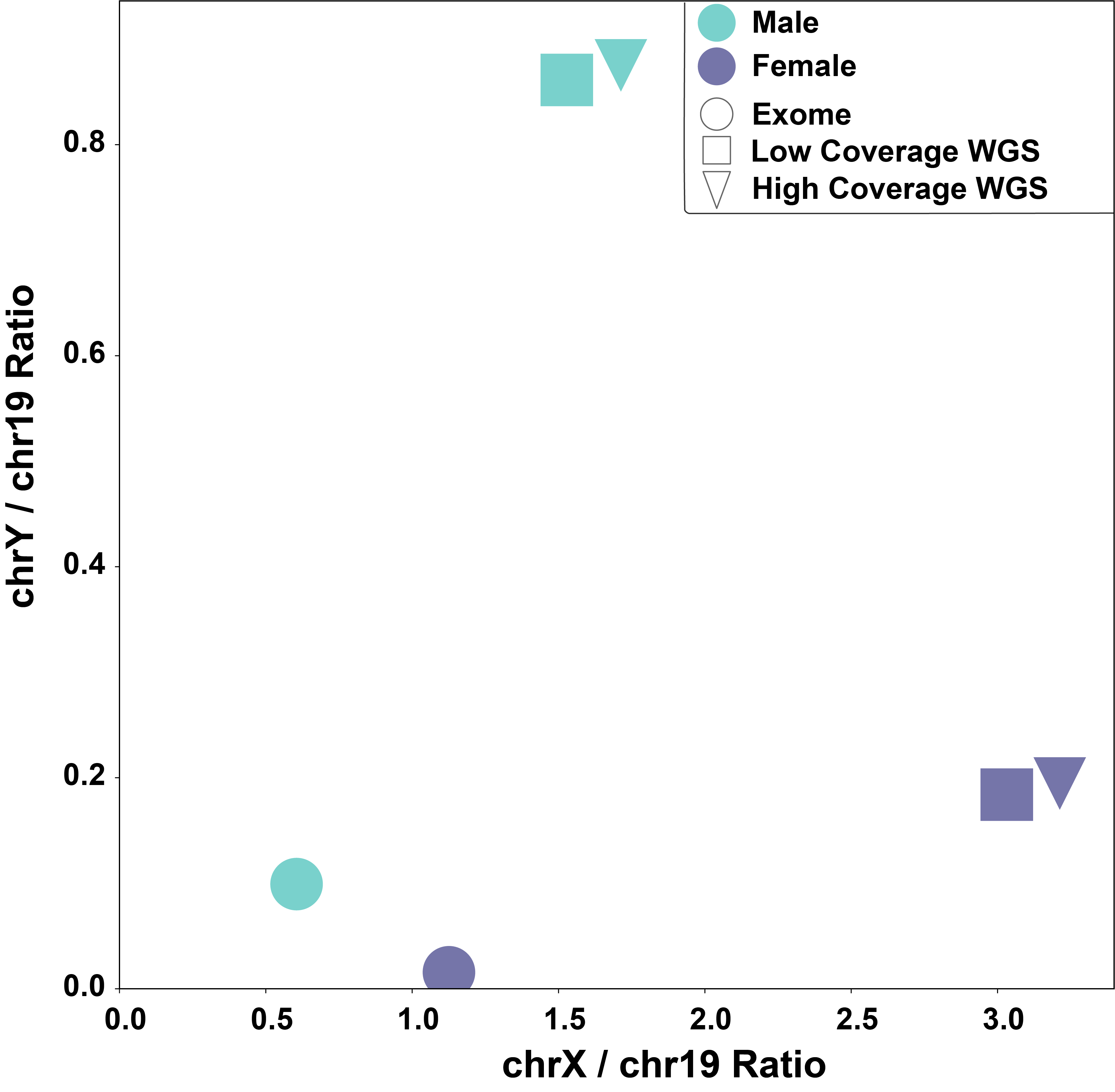
**

**Figure S8. Relative number of reads mapped to the X and Y chromosomes across different sequencing strategies.** Values of the number of reads mapped come from exome (circles), low-coverage whole-genome sequencing (squares), and high-coverage whole-genome sequencing (triangles) for a single male (green) and female (blue) individual (Dataset 1; Table S1). The number of reads mapped to chromosome 19 was used to normalize the sex chromosomes.


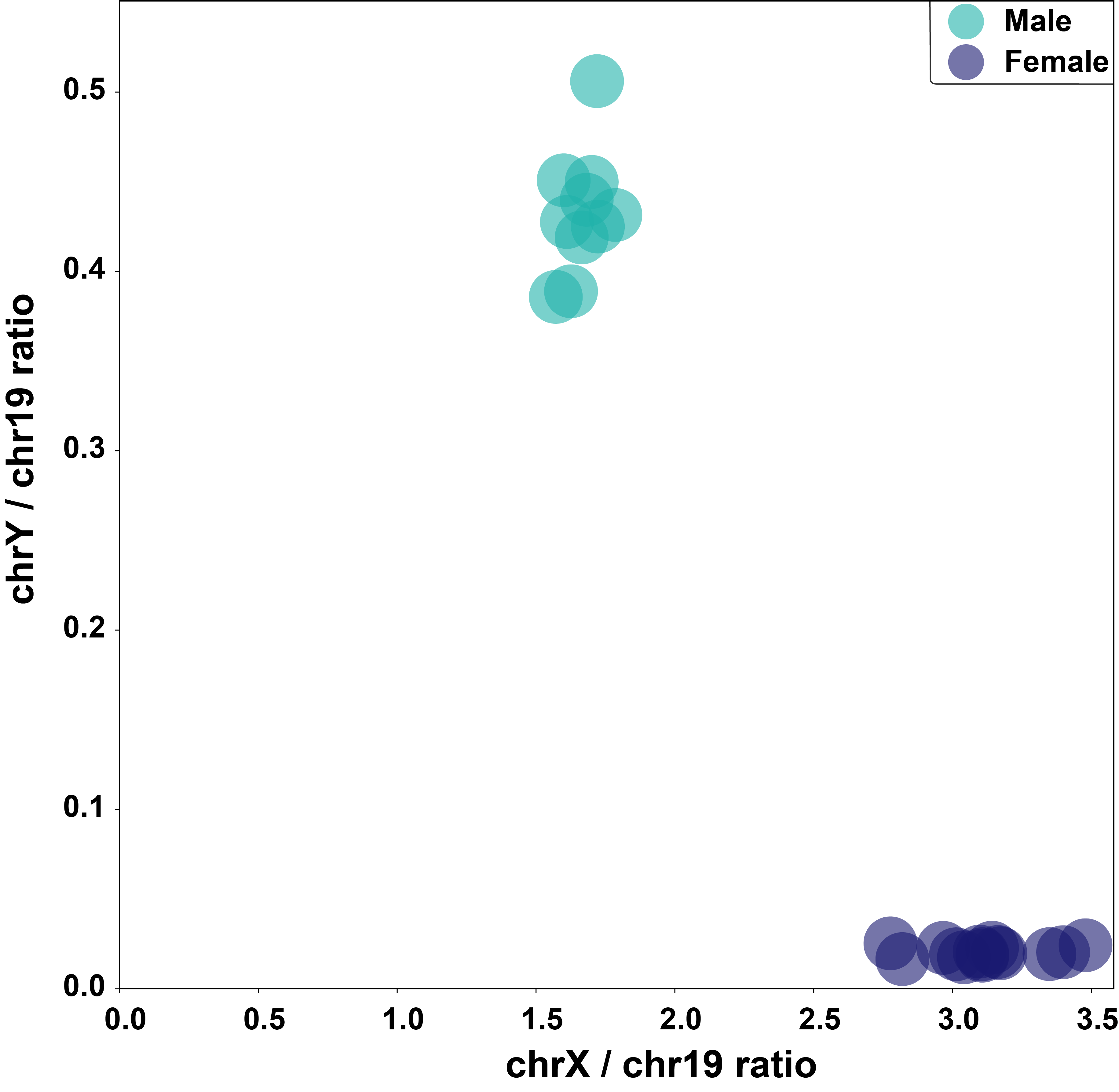


**Figure S9. Relative number of reads mapped to the X and Y chromosomes in the 1000 Genomes Project high-coverage samples.** Males are plotted in green, while females are plotted in blue (Dataset 2; Table S1). The number of reads mapped to chromosome 19 was used to normalize the sex chromosomes.
