# Supplementary material for: Identifying, understanding, and correcting technical artifacts on the sex chromosomes in next-generation sequencing data
Source: Gigascience. 2019 Jul 9;8(7):giz074. doi: 10.1093/gigascience/giz074 (PMC6615978; doi:10.1093/gigascience/giz074)
Supplement: giz074_Supplemental_Files [file giz074_supplemental_files.zip › 20190705_Webster_etal_XYalign_supplement_Gigascience.pdf]

Title:

Identifying, understanding, and correcting technical biases on the sex chromosomes in next-generation sequencing data

Authors and Affiliations:

Timothy H. Webster, Madeline Couse, Bruno M. Grande, Eric Karlins, Tanya N. Phung, Phillip A. Richmond, Whitney Whitford, Melissa A. Wilson

**Supplementary Item**	**Page**
Supplementary Methods	2
Table S1. Samples included in this study.	5
Table S2. Coordinates of major X chromosome features in hg19.	6
Table S3. Variants identified across the Y chromosome.	7
Figure S1. Read balance in XY and XX samples including fixed sites.	8
Figure S2. Read balance in ampliconic regions of the Y chromosome.	9
Figure S3. Read balance in heterochromatic regions of the Y chromosome.	10
Figure S4. Read balance in X degenerate regions of the Y chromosome.	11
Figure S5. Read balance in X-transposed regions of the Y chromosome.	12
Figure S6. Relative sequencing depth on the X and Y chromosomes in the 1000 Genomes Project high-coverage samples.	13
Figure S7. Relative mapping quality (MAPQ) on the X and Y chromosomes in the 1000 Genomes Project high-coverage samples.	14
Figure S8. Relative number of reads mapped to the X and Y chromosomes across different sequencing strategies.	15
Figure S9. Relative number of reads mapped to the X and Y chromosomes across in the 1000 Genomes Project high-coverage samples.	16

Supplementary Methods

Because we ran XYalign on many samples, we provide templates for commands here. In all cases, the exact commands are included in a Snakemake (Köster and Rahmann, 2012) pipeline available with the XYalign software distribution available at Github (https://github.com/WilsonSayresLab/XYalign). A permanent static version is deposited at Zenodo as well (Webster *et al.*, 2018).

Full XYalign pipeline on Dataset 1

We initially prepared separate XX and XY references using the following command:

```
xyalign --PREPARE_REFERENCE --ref <hg19 reference
genome> --xx_ref_out hg19.XXonly.fasta --xy_ref_out
hg19.XY.fasta --output_dir <output_directory> --
x_chromosome chrX --y_chromosome chrY --bwa_index True
```

where <hg19 reference genome> was the path to the FASTA file containing the hg19 reference, <input bam file> was a sorted BAM file, and <output directory> was the directory where XYalign wrote output. We then ran the full pipeline on all six files from Dataset 1 using the following command template:

```
xyalign --ref <hg19 reference genome> --bam <input bam
file> --output_dir <output directory> --sample_id
<sample ID> --cpus 4 --reference_mask
hg19_PAR_Ymask_startEnd.bed --window_size 5000 --
chromosomes chr19 chrX chrY --x_chromosome chrX --
y_chromosome chrY --xmx 4g --fastq_compression 4 --
min_depth_filter 0.2 --max_depth_filter 2 --xx_ref_in
hg19.XXonly.fasta --xy_ref_in ref_out hg19.XY.fasta,
```

where <sample ID> was the identification code for a given sample, hg19_PAR_Ymask_startEnd.bed was a BED file containing the genomic coordinates of the PARs in the hg19 assembly, and hg19.XXonly.fasta and hg19.XY.fasta were the two FASTA formatted reference genomes prepared in the previous step.

Variant analyses on Dataset 1

To count variants falling in major genomic regions, we intersected a BED file containing coordinates with VCF files using BEDTools (Quinlan and Hall, 2010). We first filtered VCF files using BCFtools (Li *et al.*, 2009) with the following command template:

```
bcftools filter --include 'INFO/MQ>=30 && %QUAL>=30'
<input_vcf>
```

We then identified variants unique to each file through iterations of the "subtract" command in BEDtools (Quinlan and Hall, 2010):

```
bedtools subtract -header -a <first_vcf> -b
<second_vcf>
```

Finally, in each region, we counted variants present in a given filtered VCF file using the BEDtools (Quinlan and Hall, 2010) "intersect" command:

```
bedtools intersect -c -a <BED file> -b <vcf_file>
```

where <`BED_file`> is the BED file containing genomic coordinates (Supplemental Table S2).

Inferring Genetic Sex
Next, we examined how the metrics generated by XYalign can be used to identify the sex chromosome complement of individuals from both datasets. Here, we used the CHARACTERIZE_SEX_CHROMS module of XYalign. This was automatically done for Dataset 1 when running the full pipeline (see above). For Dataset 2, we used the following command template for BAM files:

```
xyalign --CHARACTERIZE_SEX_CHROMS --ref  <1000 genomes
reference genome> --bam  <input bam file> --output_dir
<output directory> --sample_id <sample ID> --cpus 4 --
window_size 5000 --chromosomes 19 X Y --x_chromosome X
--y_chromosome Y
```

Finally, we explored the utility of the CHROM_STATS module for identifying sex chromosome complement and potentially sex-linked scaffolds with both datasets using the following command template for BAM files:

```
xyalign --CHROM_STATS --chromosomes chr1 chr8 chr19
chrX chrY chrM --bam <input_bam_1> <input_bam_2>
<input_bam_3> --ref null --sample_id
<name_of_analysis> --output_dir <output_dir>
```

We additionally ran CHROM_STATS using the above command with the addition of the "`--use_counts`" flag to calculate metrics using only the number of reads mapping to each chromosome.

We visualized all CHROM_STATS results using the plot_count_stats utility, with the command template:

```
plot_count_stats --input <chrom_stats output file> --
output_prefix <output prefix>--meta <metadata text
file> --exclude_suffix <suffix> --first_chr chrX --
second_chr chrY --const_chr chr19 --var1_marker color
--var1_marker_vals darklateblue thistle --var2_marker
```

```
shape --var2_marker_vals o s v --marker_size 1700 --
legend_marker_scale 0.4
```

where <*chrom_stats_output_file*> was either the count, mapping quality, or depth output of CHROM_STATS, <`metadata text file`> was the appropriate metadata text file, and <`suffix`> was the string to remove from filenames.

Table S1. Samples included in this study.

ID	Sex[a]	Sequencing[b]	Dataset[c]	Citation[d]
HG00512	M	E, LC, DC	Dataset 1	1
HG00513	F	E, LC, DC	Dataset 1	1
HG00419	F	DC	Dataset 2	2
NA20845	M	DC	Dataset 2	2
NA19625	F	DC	Dataset 2	2
NA19017	F	DC	Dataset 2	2
HG03052	F	DC	Dataset 2	2
HG01595	F	DC	Dataset 2	2
NA18525	F	DC	Dataset 2	2
NA20502	F	DC	Dataset 2	2
HG02568	F	DC	Dataset 2	2
NA18939	F	DC	Dataset 2	2
HG03642	F	DC	Dataset 2	2
HG00759	F	DC	Dataset 2	2
HG01112	M	DC	Dataset 2	2
HG01583	M	DC	Dataset 2	2
HG01051	M	DC	Dataset 2	2
HG00268	F	DC	Dataset 2	2
HG03742	M	DC	Dataset 2	2
NA19648	F	DC	Dataset 2	2
HG00096	M	DC	Dataset 2	2
HG02922	F	DC	Dataset 2	2
HG01565	M	DC	Dataset 2	2
HG01879	M	DC	Dataset 2	2
HG01500	M	DC	Dataset 2	2
HG03006	M	DC	Dataset 2	2

[a]Reported sex.

[b]Sequencing strategy: exome (E), low-coverage whole-genome (LC), and deep-coverage whole-genome (DC).

[c]Dataset membership for this study.

[d]1. The 1000 Genomes Project Consortium, 2015. 2. Sudmant *et al.*, 2015. References in main text.

Table S2. Coordinates of major X chromosome features in hg19.[a]

Chromosome	Start	End	Feature[b]
chrX	60000	2649520	PAR1
chrX	2649520	58632012	XAR
chrX	61632012	88395830	XCR1
chrX	88395830	92683067	XTR
chrX	92683067	154931044	XCR2
chrX	154931044	155260560	PAR2

[a]Coordinates in zero-index, half open format (i.e., bed format).

[b]PAR1: pseudoautosomal region 1; XAR: X-added region; XCR: X-conserved region; XTR: X-transposed region.

Table S3. Variants identified across the Y chromosome.[a]

Regions[b]	Variant sites passing filters[c]	Mean read balance
Ampliconic	2606	0.39
Heterochromatic	4475	0.36
Other	168	0.49
Pseudoautosomal	15	0.82
X degenerate	1262	0.75
X transposed	752	0.57

[a]Variant sites (sites with a non-reference allele) identified on the Y chromosome in whole-genome sequencing data from the XY individual in Dataset 1

[b]Regions from Poznik et al. 2013

[c]Depth >= 4, GQ >= 30, QUAL >= 30

[d]Read balance at a given site is defined as the number of reads containing a non-reference allele divided by the total number of reads mapped to a site.

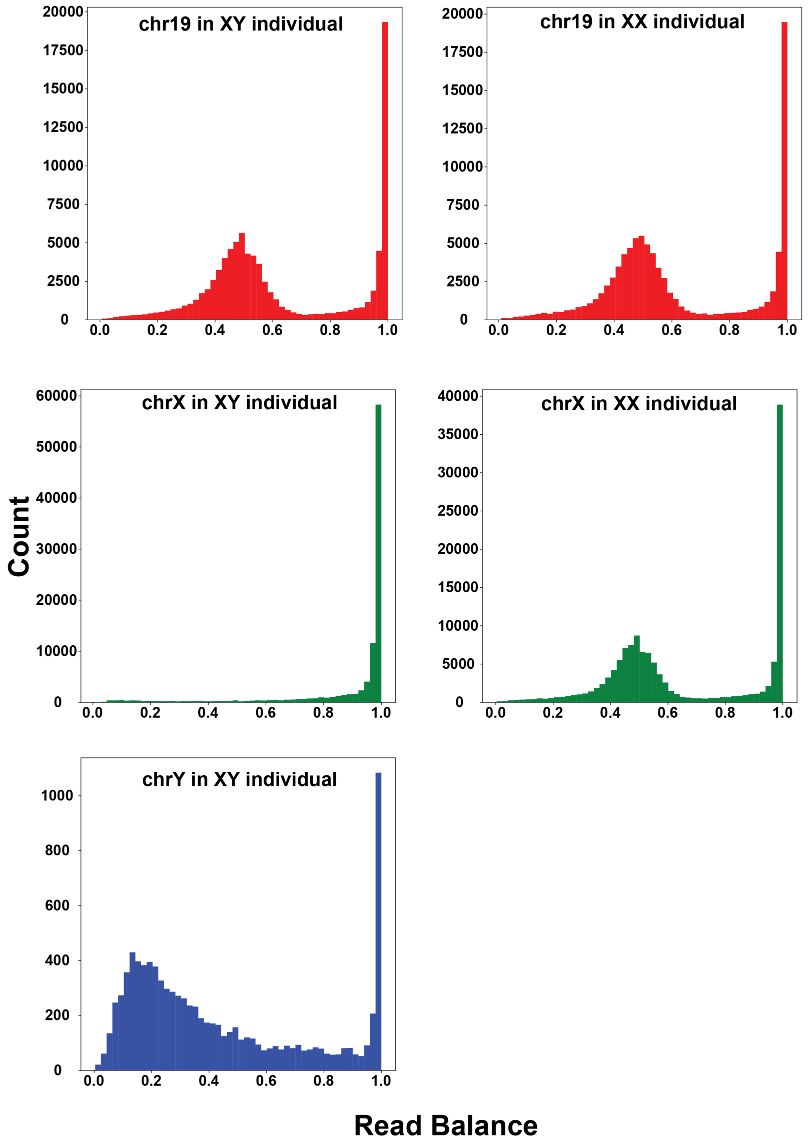

Figure S1. Read balance in XY and XX samples including fixed sites. Histograms of read balance for an XY sample (Left Column; A, C, and E) and XX sample (Right Column; B and D) from Dataset 1 across chromosome 19 (Top; A and B), chromosome X (Middle; C and D), and chromosome Y (Bottom; E). Read balance at a given site is defined as the number of reads containing a non-reference allele divided by the total number of reads mapped to a site. The full distribution of read balances is presented.

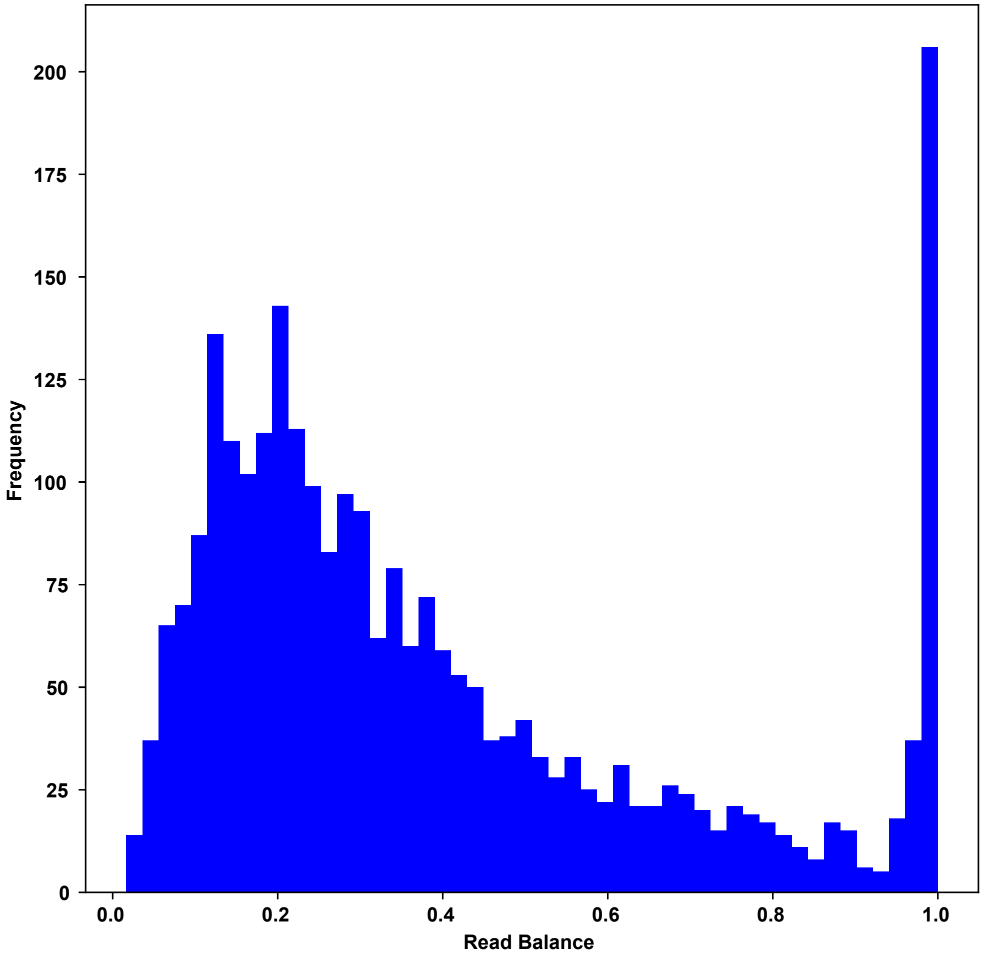

Figure S2. Read balance in ampliconic regions of the Y chromosome. Histogram of read balance for an XY sample from Dataset 1 across Y chromosome ampliconic regions. Read balance at a given site is defined as the number of reads containing a non-reference allele divided by the total number of reads mapped to a site. The full distribution of read balances is presented.

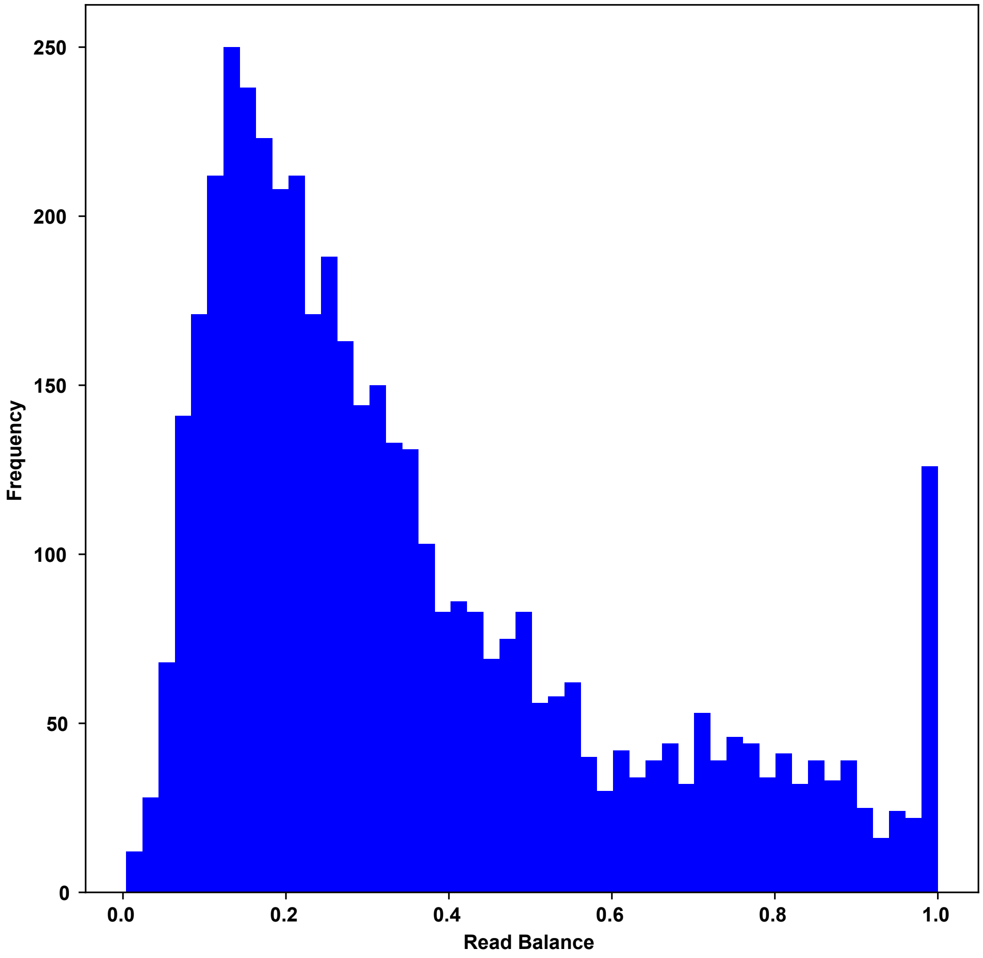

Figure S3. Read balance in heterochromatic regions of the Y chromosome. Histogram of read balance for an XY sample from Dataset 1 across Y chromosome heterochromatic regions. Read balance at a given site is defined as the number of reads containing a non-reference allele divided by the total number of reads mapped to a site. The full distribution of read balances is presented.

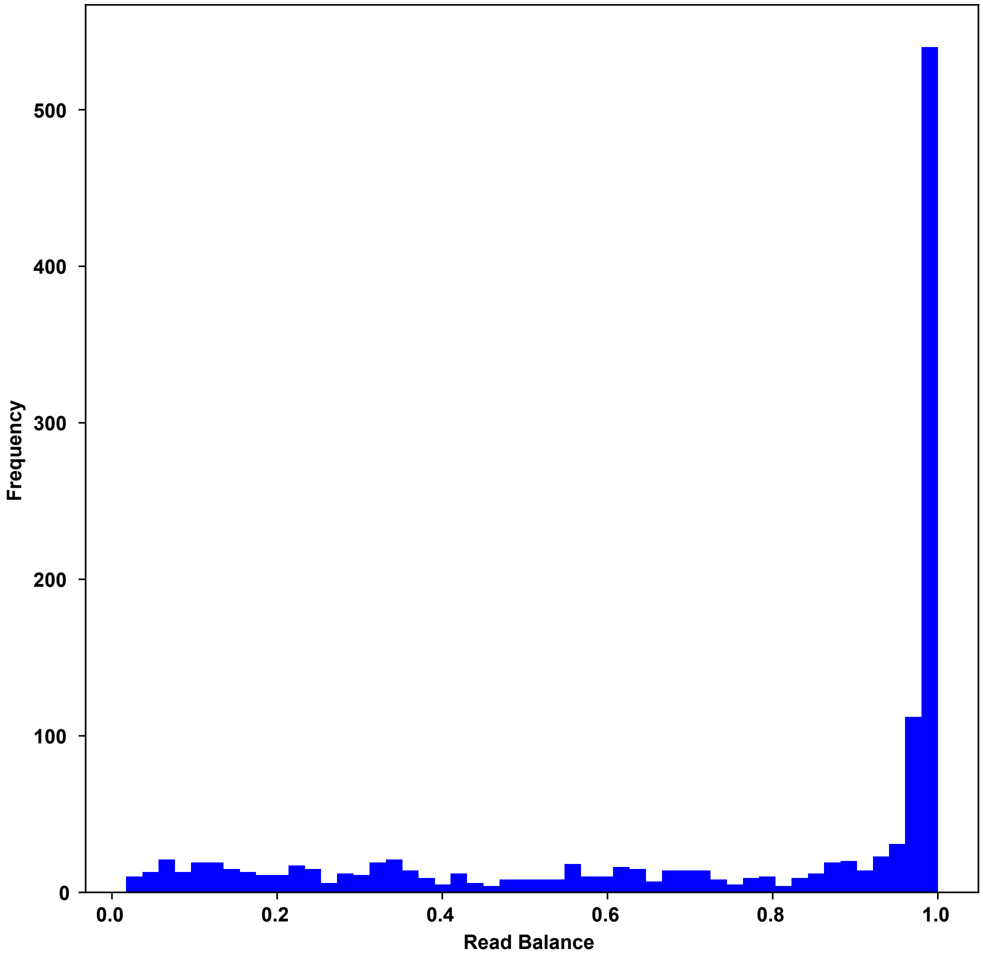

Figure S4. Read balance in X degenerate regions of the Y chromosome. Histogram of read balance for an XY sample from Dataset 1 across X degenerate regions of the Y chromosome. Read balance at a given site is defined as the number of reads containing a non-reference allele divided by the total number of reads mapped to a site. The full distribution of read balances is presented.

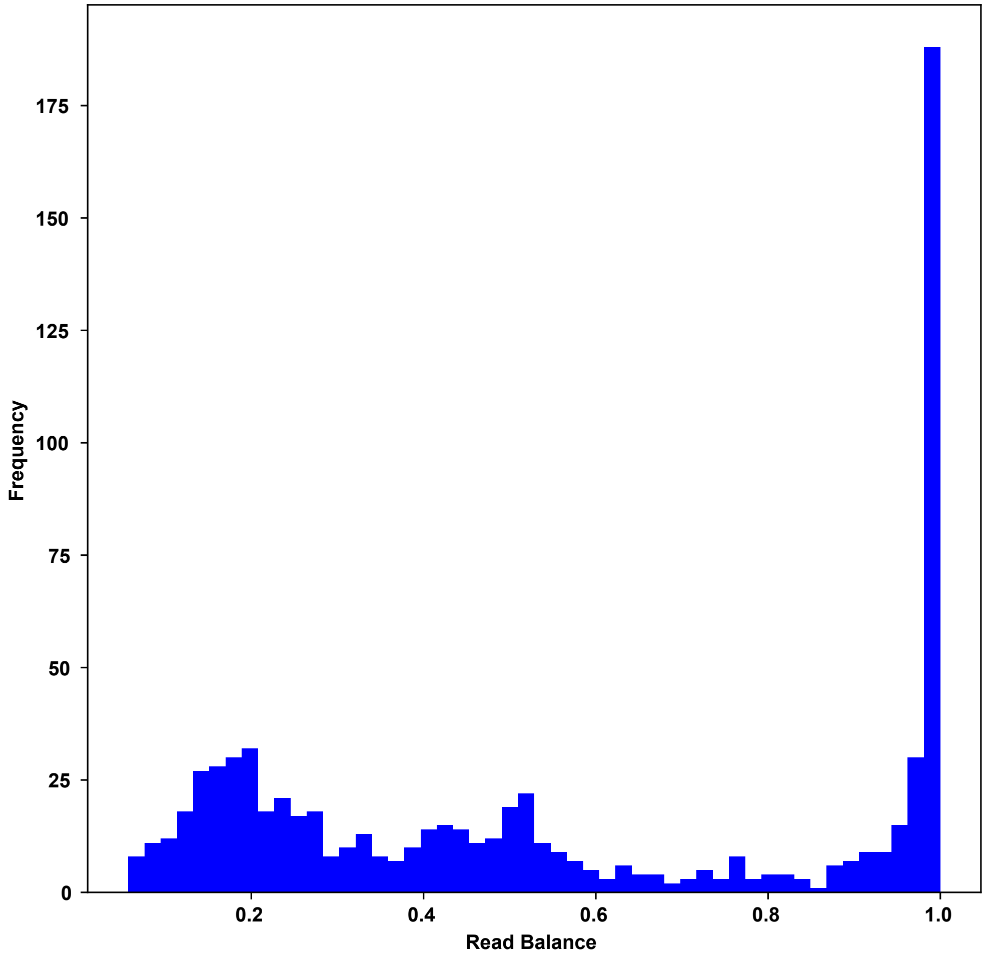

Figure S5. Read balance in X-transposed regions of the Y chromosome. Histogram of read balance for an XY sample from Dataset 1 across X-transposed regions of the Y chromosome. Read balance at a given site is defined as the number of reads containing a non-reference allele divided by the total number of reads mapped to a site. The full distribution of read balances is presented.

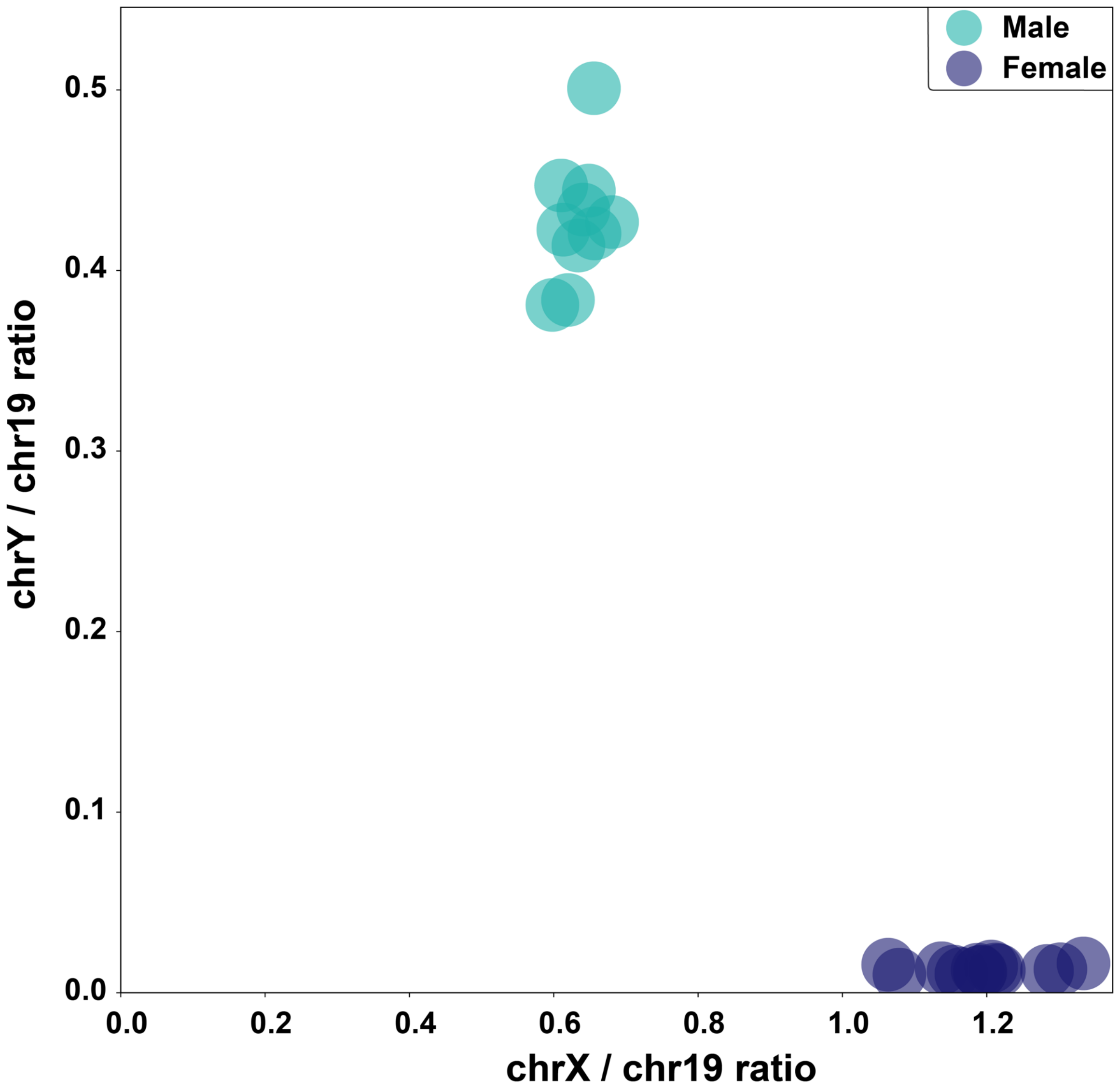

Figure S6. Relative sequencing depth on the X and Y chromosomes in the 1000 Genomes Project high-coverage samples. Males are plotted in green, while females are plotted in blue (Dataset 2; Table S1). Mean depth on chromosome 19 was used to normalize the sex chromosomes.

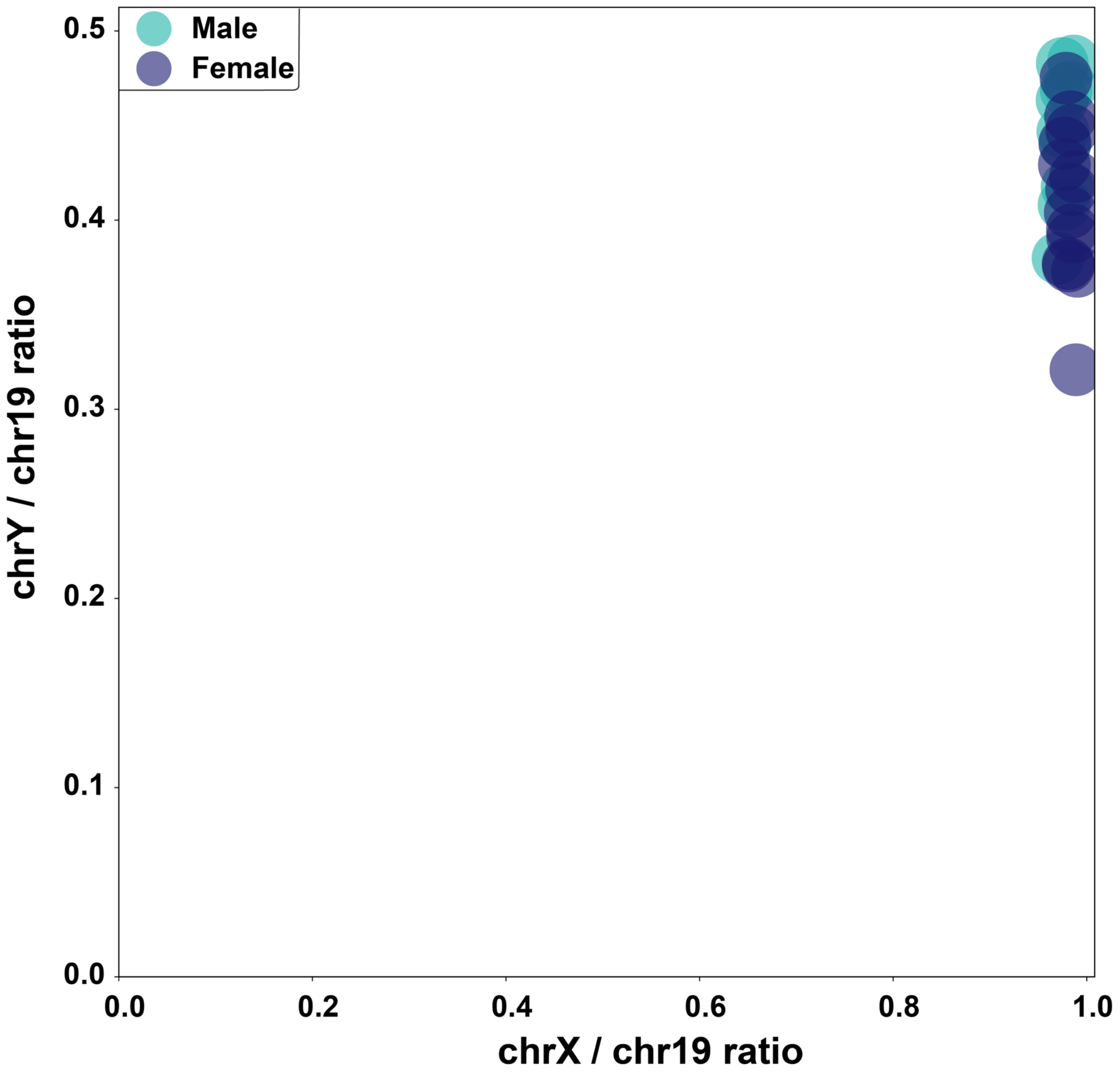

Figure S7. Relative mapping quality (MAPQ) on the X and Y chromosomes in the 1000 Genomes Project high-coverage samples. Males are plotted in green, while females are plotted in blue (Dataset 2; Table S1). Mean MAPQ on chromosome 19 was used to normalize the sex chromosomes.

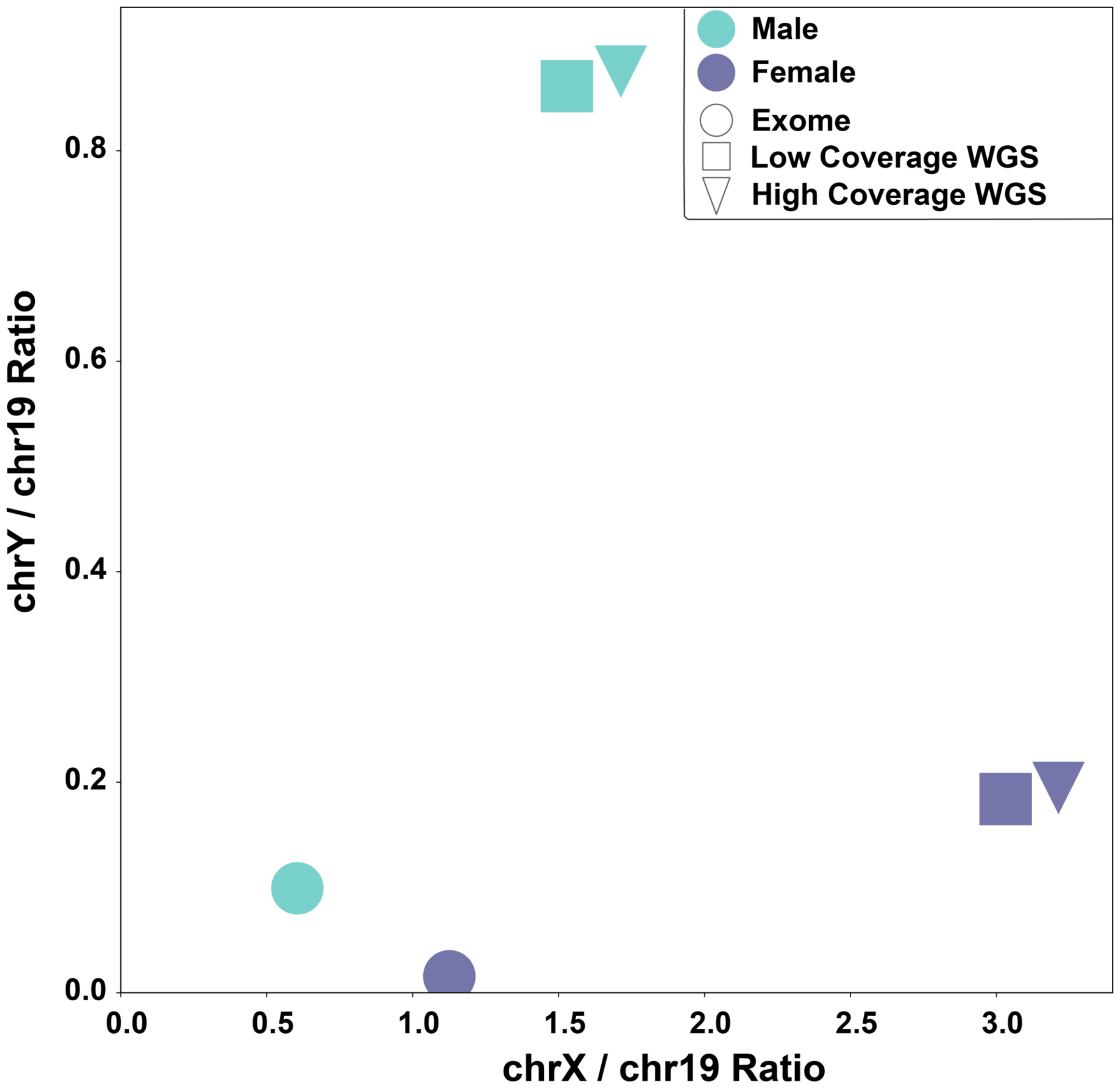

Figure S8. Relative number of reads mapped to the X and Y chromosomes across different sequencing strategies. Values of the number of reads mapped come from exome (circles), low-coverage whole-genome sequencing (squares), and high-coverage whole-genome sequencing (triangles) for a single male (green) and female (blue) individual (Dataset 1; Table S1). The number of reads mapped to chromosome 19 was used to normalize the sex chromosomes.

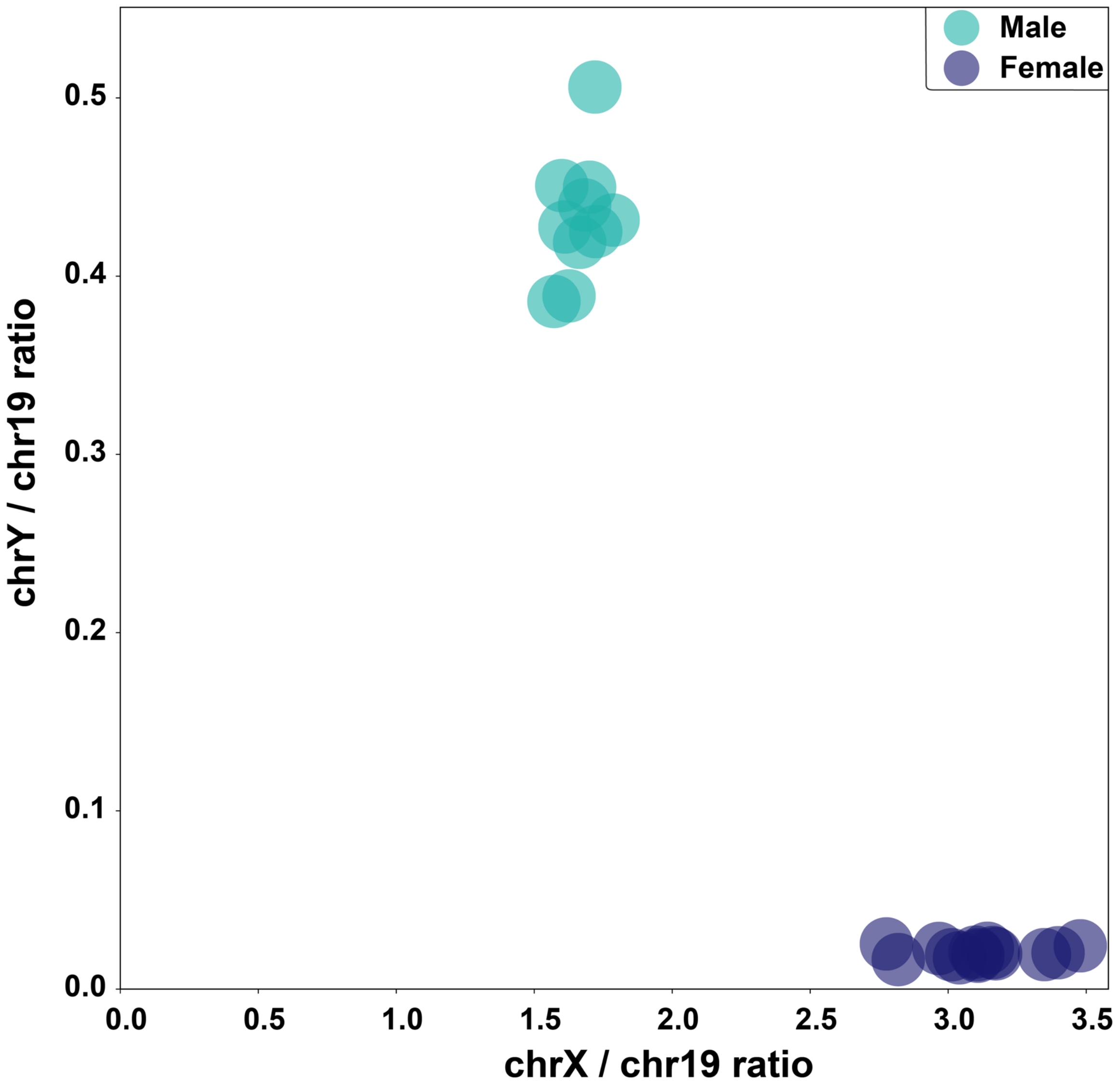

Figure S9. Relative number of reads mapped to the X and Y chromosomes in the 1000 Genomes Project high-coverage samples. Males are plotted in green, while females are plotted in blue (Dataset 2; Table S1). The number of reads mapped to chromosome 19 was used to normalize the sex chromosomes.
