# Supplementary material for: Identifying, understanding, and correcting technical artifacts on the sex chromosomes in next-generation sequencing data
Source: Gigascience. 2019 Jul 9;8(7):giz074. doi: 10.1093/gigascience/giz074 (PMC6615978; doi:10.1093/gigascience/giz074)
Supplement: giz074_Response_to_Reviewer_Comments_Original_Submission [file giz074_response_to_reviewer_comments_original_submission.pdf]

# Author's Response To Reviewer Comments

Close

December 3, 2018

Dear Dr. Hans Zauner and Editors of Gigascience,

We are resubmitting our manuscript, GIGA-D-18-00312, titled “Identifying, understanding, and correcting technical artifacts on the sex chromosomes in next-generation sequencing data.” Please note that this title has changed slightly, as we now use the word “artifacts” instead of “biases.”

We have revised our manuscript following the, what we feel, were very helpful comments we received from you and the reviewers. We describe these changes below in our point-by-point responses to comments.

Thank you very much for allowing us the opportunity to revise our manuscript.

Sincerely,
Timothy H. Webster and Melissa A. Wilson Sayres

Editor Comments:

1) It is not entirely clear whether the tool provides a solution for problems that cannot be solved with already available tools. Our author guidelines for Technical Notes say that the tool needs to show innovation in the approach "or have added benefits that have been needed in this arena". The justification for this need, in light of available tools, should be improved in a revised version (see the comments of reviewer 1).

--Our goal is that this manuscript serves two purposes: (1) identify for the first time, to our knowledge, what appears to be a major source of technical artifacts in next-generation sequencing data; and (2) present a tool to help identify, understand, and correct these biases. So, we hope to first emphasize the importance of this manuscript highlighting the problems caused by sex chromosome mismapping, as these are likely present in every next-generation sequencing study of an organism with heteromorphic sex chromosomes, even for species whose assemblies are missing the Y (XX/XY system) or W (ZZ/ZW) chromosomes.

In addition, while XYalign does wrap a variety of packages and programs to carry out some of its functionality, we emphasize (1) that the purpose of XYalign is to provide a clear, tested, reproducible solution to sex chromosome mapping, and (2) though it is possible to undertake some of these steps by combining available tools, to our knowledge no one has done so. We feel (1) in particular is consistent with GigaScience’s emphasis on best practices and reproducibility. With this in mind, we view the software XYalign as meeting the “added benefits that have been needed in this arena” criteria: the problem we describe is and will continue to be pervasive, and there is a need for a clear, reproducible, and easily implemented software solution to understand and address it. Further, (2) addresses innovation in the approach, if even a measured and conceptual innovation that has not yet been implemented in current tools.

To help clarify the problem we are addressing and its importance, we updated the abstract, which now reads:
“Mammalian X and Y chromosomes share a common evolutionary origin and retain regions of high sequence similarity. Similar sequence content can confound the mapping of short next-generation sequencing reads to a reference genome. It is therefore possible that the presence of both sex chromosomes in a reference genome can cause technical artifacts in genomic data and affect downstream analyses and applications. Understanding this problem is critical for medical genomics and

population genomic inference. Here, we characterize how sequence homology can affect analyses on the sex chromosomes and present XYalign, a new tool that: (1) facilitates the inference of sex chromosome complement from next-generation sequencing data; (2) corrects erroneous read mapping on the sex chromosomes; and (3) tabulates and visualizes important metrics for quality control such as mapping quality, sequencing depth, and allele balance. We find that sequence homology affects read mapping on the sex chromosomes and this has downstream effects on variant calling. However, we show that XYalign can correct mismapping, resulting in more accurate variant calling. We also show how metrics output by XYalign can be used to identify XX and XY individuals across diverse sequencing experiments, including low and high coverage whole genome sequencing, and exome sequencing. Finally, we discuss how the flexibility of the XYalign framework can be leveraged for other uses including the identification of aneuploidy on the autosomes. XYalign is available open source under the GNU General Public License (version 3)."

2) I agree with reviewer 2 that the structure of your manuscript is rather confusing because "descriptions, user recommendations, general notes, and cross referencing other parts of the paper, are interleaved in the text" (reviewer 2).

Please revise the paper to more clearly separate the different aspects. In this respect, you don't need to strictly stick to our "instructions" regarding article sections (such as "Results" and "Discussion") and you can introduce your own sections - e.g. "overview of the tool", "comparison with existing solutions", "user recommendations", "case studies", etc. , whatever makes sense.

I'm not sure I entirely agree with referee 2 regarding their recommendation to move material to the supplement. We don't have space restrictions. I think it's useful to have most of the information in the paper itself, as long as the reader is guided better, by more clearly separating the different topics. However, command lines and parameters may be indeed better placed in the supplement as a text file, also to make them easier to re-use.

--We now lead with a shorter "Methods" section that describes the data and the analyses used in the paper. We then follow with a separate "Software Description and Implementation" section. Further, in the new Methods section, we briefly describe our data and analyses, and have moved our more detailed description of methods with command templates to a new supplementary section called "Supplementary Methods." We note that, overall, the content of these sections is virtually the same; we have only changed their structure.

3) Please also be aware that at GigaScience we put a lot of emphasis on full reproducibility. I note you provide the tool via GitHub and Zenodo, which is great. Please also consider to share data and code via the CodeOcean reproducibility platform (https://codeocean.com/). We collaborate with Code Ocean, and if you cite your code ocean DOI in the paper, we can integrate this in our own repository GigaDB.

--After exploring CodeOcean, we do not believe that it can sufficiently handle our dataset and analyses with only 10 free hours and 20 GB of storage. Rather, we highlight that, more than just providing the tool in those repositories, the full Snakemake pipeline and Conda environment are available on Github and Zenodo as well.

4) Please also ensure that your revised manuscript conforms to the journal style, which can be found in the Instructions for Authors on the journal homepage. In particular, please use numbered referencing.

--We have changed citations and used double spaces to match the journal style.

5) Please register any new software application in the SciCrunch.org database to receive a RRID (Research Resource Identification Initiative ID) number, and include this in your manuscript. This will facilitate tracking, reproducibility and re-use of your tool.

--We have registered XYalign with SciCrunch and have added its RRID (SCR_016661) to the manuscript (line 179).

Reviewer reports:

Reviewer #1: This paper presents a tool to deal with sex chromosomes, which are distinct from autosomes as the X- and Y- linked alleles (called gametologs) are usually very divergent from each

other comparing to the two alleles of autosomes. This tool has two usages: 1) mask the already identified X or Y chromosome sequences for later more accurate calling of variants and coverage data 2) produce metrics and plots for inferring the X- or Y- linkage of unclassified sequences. I acknowledge the importance of the treatment of 1) for downstream analyses of variant calling. However, the scientific or technical novelty presented by this work, and also the scope of application are limited. Both 1) and 2) can be accomplished by all other available routine bioinformatic softwares. For 1), it needs a well-assembled genome like that of human, with X and Y sequences and also their PARs separately assembled as one sequence per chromosome. This is very rare for most species, and also PAR sequences are usually collapsed as one sequence instead of two in these species. For 2), the difficulty usually lies in the 'long-tail' problem, i.e., unclassified scaffold sequences that do not show an expected female vs. male ratio of depth as 0.5 for the X, and as approaching 0 for the Y. This tool does not offer new statistical methods to discriminate scaffolds whose coverage fall in between expectations of sex chromosome and autosome, but just provide their distribution.

• Novelty

We feel that our work is novel in several ways. (1) To our knowledge, no one has previously attempted to describe the full scope of the effect of sex chromosome homology on downstream analyses using high throughput sequencing data (i.e., variant calling). (2) We have never seen anyone propose the complete masking of the Y (W) chromosome in XX (ZZ) individuals or evaluate its utility. (3) We have never seen anyone compare and evaluate different methods for inferring genetic sex across sequencing strategies (e.g., exome vs. low-coverage vs. high-coverage). (4) While much of XYalign's functionality involves wrapping external tools in certain combinations and with specific parameter sets, a large portion is novel code written in Python and thus is not currently possible using other tools. Moreover, while it certainly is possible to run analyses similar to those in XYalign that involve wrapping external tools, to our knowledge no tool combines all necessary steps in a robust and reproducible manner. And importantly, no one has run many of these steps using other tools, even if they possible. We also suggest that providing a simple option to reproducibly undertake these methods will improve transparency, reproducibility, and data/result comparability in genomic analyses (e.g., 10 studies generating sex-specific reference assemblies with the same XYalign command line versus 10 studies independently writing code to do the masking; there are many small steps in the process, and missing or changing one could alter downstream analyses in a variety of ways).

• Scope of application

We describe two uses for XYalign in detail in the manuscript (there are more, however, and we list some additional uses in the section titled “Additional Uses” starting on line 471): (1) understanding and correcting artifacts stemming from sex chromosome homology, and (2) inferring genetic sex. For 1, both sex chromosome sequences are needed in the assembly, but no other information is required (PARs, etc.)—just knowledge of the set of sex chromosome scaffolds. While this is not the most common state, it's not “very rare.” In the manuscript, we list a variety of example organisms for which this is the case (line 436: “e.g., human, chimpanzee, rhesus macaque, gorilla, mouse, rat, chicken, Drosophila”). This is not an exhaustive list, and the list will rapidly increasing with new efforts such as the Vertebrate Genomes Project (https://vertebrategenomesproject.org/phase-one/), which aims to generate a reference using the heterogametic sex across many vertebrate species. However, even limiting scope to the list of species we provide, there are still at least hundreds of genomic studies of humans, nonhuman primates, rodents, and Drosophila that involve the sex chromosomes, with likely many more to come. For 2 (inferring genetic sex), we highlight that we do not simply evaluate depth of coverage. We further show how mapping quality, read balance, and population variation can be used as well. These data can be used in any number of species, with or without reference genomes.

• XYalign data needs

As mentioned above, XYalign neither requires a high quality reference nor PAR annotation. In the text, we discuss PAR annotation as conditional (note the “if”): (line 467: “if pseudoautosomal regions are present in the reference genome, they should be masked in the heterogametic sex's assembly output by the PREPARE_REFERENCE module.”)

• Methods for inferring sex

We provide a variety of methods for inferring sex and often present the distributions because we anticipate thresholds will vary based on sex chromosome ploidy and sequence divergence. The latter varies substantially across taxa. Further, as we show, specific thresholds will vary depending on the

sequencing approach used (e.g., low coverage whole genome, high coverage whole genome, or exome capture).

I have some detailed comments below:
1) page 5, please clarify what does 'secondary and supplementary read mapping' here mean?

--We updated the sentence to read: “During traversal, depth is calculated as the total length of all reads (primary alignments only) mapping to a genomic window divided by the total length of the window.” (line 230)

2) page 9, I am not sure if we should call these patterns 'artifacts' as they are clearly biologically meaningful. For example, a reduction of MAPQ for the X-transposed region.

--We changed this sentence to “We found that sex chromosome sequence homology leaves a variety of detectable signals in the genome” (line 291).

However, we do use “artifacts” throughout much of the rest of the manuscript because while, for example the lower MAPQ in the XTR is a result of a real biological phenomenon, MAPQ is only lower in this region (and able to affect variant calling) because both the X and Y chromosomes are included in the assembly (a technical/methodological issue).

3) In figure 1, the depths of PAR, ampliconic regions, are reduced comparing to the rest of the X chromosome, in both A and B plot. It should be clarified, as they are reduced for different reasons. I assume that for PAR, it is because half of the reads were mapped to the other sex chromosome, while for ampliconic regions, it was affected by less uniquely mapped reads comparing to other regions?

--In the text, we only broadly discuss the changes between using a standard (XY) and sex-specific reference in an XX individual, which are summarized in 1D. Most striking are the increases in depth in PAR1, XTR, and PAR2. However, other regions, including ampliconic regions, also exhibit increases in depth as well, suggesting that reads in those regions also may have been mapping to the Y chromosome

4) the MAPQ value of figure 2A is approaching 0, any explanations?

--We are unsure what this question is referring to. In Figure 2A, 60 (the maximum MAPQ for BWA MEM) is the mode, with only a few small regions approaching 0, mostly in PAR1, PAR2, and ampliconic regions (see Figure 2C for regions).

5)page 13, 'the Y chromosome of the XX individual', please clarify what do you mean here?

--We changed the text to read: “Furthermore, XX individual no longer had any variant calls or mapped reads on the Y chromosome...” (line 664). This sentence is simply describing that the XX individual had Y chromosome mapped reads and variant calls when using a default reference genome mapping approach, but not after XYalign variant calling. A lack of Y chromosome data is consistent with the biological state of the individual (i.e., an XX individual does not have Y chromosome sequence).

6) page 15, can you please check where are the reads of 0.2 peak coming from on the Y?

--We explored a variety of possible causes of this peak, but could not explain it. We discuss this in text on line 390: “We observed one exception to this pattern: the Y chromosome exhibited a peak around 0.2 in addition to the one near 1.0 (Figure 4). All variants included in analyses met thresholds for depth, site quality, and genotype quality, so quality does not appear to be a driving factor of this pattern. This pattern also remained after genomic windows of low mapping quality and irregular depth were removed. We are currently unable to explain these results and more work is thus required to understand the factors responsible for this pattern and whether similar results are obtained on the W chromosome in ZW systems.”

Reviewer #2:
SUMMARY

The authors describes a software XYalign that, in summary, aims to correct misalignments of reads originating from the sex chromosomes. The authors shows that incorrect alignments on the sex chromosomes is common in certain regions, and they occur due to the high sequence similarity between

the sex chromosomes, together with the fact that the reference genome does not reflect the structure of the individual (either XX or XY). Their results also indicates that improving the alignments helps downstream variant calling on the sex chromosomes.

XYalign seems to solve a practical problem that the authors encountered, and the software is well documented in terms of installation and user instructions. The introduction gives a clear description of what the aim with the paper. I however find the method and result sections difficult to follow. My assumption is that this is because method descriptions, user recommendations, general notes, and cross referencing other parts of the paper, are interleaved in the text. There are also a lot of parameter settings and command lines given in between text that, in my opinion, could be moved to supplementary to improve the flow of the text. Specific comments below.

MAJOR

* Methods section
- The methods section needs a more formal writing that only focuses on describing the methods. This will help readability and also allow assessment of the methods and to adress any concerns in the analysis. I suggest providing either with pseudocode or text that minimally describe, in detail, the _input_, _workflow_, and _output_ for each of the six modules. This should be presented without including any comments about alternative use cases, comparison to other modules, and other notes (see e.g. "CHROM_STATS" and "CHARACTERIZE_SEX_CHROMOSOMES"). In my opinion, such notes should come after the method is presented.

--We now lead with a shorter “Methods” header that describes the data and the analyses used in the paper. We then follow with a separate “Software Description” section. Further, in the new Methods section, we briefly describe our data and analyses, and have moved our more detailed description of methods with command templates to a new supplementary section called “Supplementary Methods.” We note that, overall, the content of these sections is virtually the same; we have only changed the structure of these sections.

- After "ANALYZE_BAM" comes a description of "XYalign" (the main program), and the text jumps back to "CHARACTERIZE_SEX_CHROMOSOMES". It makes it difficult to follow the structure.

--Our software description first walks through each module, and then discusses the full pipeline. We believe this is critical because, in most cases, we anticipate researchers using individual modules rather than the full pipeline (discussed in “Recommendations for researchers” in the Discussion). To help clarify, we added section headers under Software Description, including “Implementation”, “Modules”, “Full Pipeline” and “Operation”. These headers split the sections Reviewer 2 describes here.

- Most of the "use cases" section should be moved to supplementary to improve readability. The first paragraph in this section seems to describe the datasets, and could be labelled datasets, and then a quick summary of what was analyzed could be given without providing command lines and text describing specific argument settings.

--We have moved all command templates to the supplementary materials, and now describe the data and analyses more briefly in the main Methods section.

* Result section
- Which are the datasets presented in the figures 1-4 and table 1 in the results section? Is it averages across 24 individuals, or only one individual? Provide information if it's Dataset 1 or 2 that is being used.

--We have added this information to the figure legends, and as a footnote in the table.

- If dataset 1 and 2 are being separately analyzed, maybe split the results section up into the two different use cases to clarify and separate the message of the separate analysis.

--We have split the methods and results/discussion (with subheadings) to reflect the two different sets of analyses: (1) sequence homology and effects on downstream analyses (dataset 1); (2) inferring genetic sex (dataset 1 and dataset 2).

- Dataset 1: What is the reason behind using exome, low-coverage, and deep coverage data of two individuals? Is it to show that XYalign can work with all these datasets? Which data does is most suitable for XYalign? I see a couple of sentences about this in "inferring genetic sex" but please sharpen the message.

--We added the following text (line 132): "We used the high-coverage whole-genome sequencing data from Dataset 1 to identify and understand the effects of sex chromosome homology on genomic data and analyses. We used the full Dataset 1 to observe if patterns of depth and mapping quality can be used to identify genetic sex in a similar way across sequencing strategies (exome, low-coverage whole-genome, and high-coverage whole genome). Finally, we used Dataset 2 to test whether population data can be easily used to identify the genetic sex of individuals."

- Table 1. False positives and true negatives are used as labels of the variant calls. This is an overstatement unless the calls are actually validated. If no such additional validation is performed, I suggest to not use this labelling. Either provide an external evaluation of (at least a larger subset of) the calls to give evidence that these are in fact true positives/false negatives, or use other labels, e.g. the actual data presented: "variant calls present before/after masking".

--We removed false positives and negatives, and we now use references to "before" and "after" masking (Table 1).

- Under my assumption that Table 1 is data from one individual, I think the manuscript would be significantly strengthened if the authors, found a way to present the results for the 24 individuals combined. For example, maybe variant calls could be compared across individuals e.g. in terms of coordinates. This would be one example of an attempt of validation of the variant calls (related to previous point with false and true calls).

--For Dataset 1, we were able to access raw data and assemble from scratch, allowing a before/after comparison with everything equal (i.e., all software, parameters, etc.) except for the reference (i.e., no masking vs. masking). We included the 24 individuals from Dataset 2 to both test XYalign's genetic sex inference on a population sample as well as on data assembled elsewhere. As such, a before/after comparison would not be comparable because of our inability to exactly match original software, etc. used during original assembly when we undertook remapping steps. We therefore opted to focus on the data that we were able to fully control (Dataset 1) for all analyses involving sex chromosome homology (including variant analyses).

MINOR:

* Provide reason to: "If pseudoautosomal regions (PARs) are present on both sex chromosome sequences in the reference, we strongly suggest masking the PARs on the Y chromosome, allowing reads from these regions to map exclusively to the X chromosome in XY individuals." If two copies are present (onion X and one on Y), wouldn't it be better to keep two copies when mapping to avoid coverage bias? Maybe I'm misinterpreting something here.

--The PARs present the most extreme form of the sequence bias we are highlighting in this manuscript (identical sequence present on both sex chromosomes). By including both PARs, mapping quality will reduce to zero (Figure 2A), leading to an inability to confidently call variants on the PAR in either chromosome (Table 1). Instead, by masking the Y chromosome PARs, all reads will map to the X chromosome PARs, allowing for variant calling similar to that of the autosomes.

While this topic is covered throughout the manuscript, we specifically discuss this with respect to the PARs on line 84:

"A reference genome that includes the entire sequence content from both sex chromosomes will thus duplicate the PARs and substantially reduce mapping quality in these regions because most reads will identically map to two regions in the reference assembly. This stands in contrast to autosomal sequence, for which each diploid autosome is represented just once in the reference genome."

Close
