# Supplementary material for: Identifying, understanding, and correcting technical artifacts on the sex chromosomes in next-generation sequencing data
Source: Gigascience. 2019 Jul 9;8(7):giz074. doi: 10.1093/gigascience/giz074 (PMC6615978; doi:10.1093/gigascience/giz074)
Supplement: giz074_Response_to_Reviewer_Comments_Revision_1 [file giz074_response_to_reviewer_comments_revision_1.pdf]

Author's Response To Reviewer Comments

Close

April 10, 2019

Dear Dr. Hans Zauner and Editors of Gigascience,

We are resubmitting our revised manuscript, GIGA-D-18-00312, titled “Identifying, understanding, and correcting technical artifacts on the sex chromosomes in next-generation sequencing data.” Please note that this title is the same as that of our first resubmission, and slightly different from our first submission, as we now use the word “artifacts” instead of “biases.”

We present point-by-point responses to reviewer comments below. To distinguish our responses from reviewer text, we have placed our responses between blocks of "###" and placed a "*" at the beginning of every reviewer paragraph/comment.

Thank you very much for allowing us the opportunity to revise our manuscript.

Sincerely,
Timothy H. Webster and Melissa A. Wilson

Editor Comments:

*Your revised manuscript "Identifying, understanding, and correcting technical artifacts on the sex chromosomes in next-generation sequencing data" (GIGA-D-18-00312R1) has been re-assessed by our two reviewers.

*As you will see from their reports below, opinions are a bit mixed: While reviewer 2 is generally happy (pending some minor additional comments), reviewer 1 is rather unconvinced regarding the novelty and potential use cases of your method.

*On balance, I feel your manuscript is suitable for further consideration as a "Technical Note", but I agree with the reviewer in so far as it will be helpful to address the latest concerns in the manuscript, and discuss some of the context the reviewer mentions. (in addition to addressing the specific additional points made by reviewer 2).

###
Thank you for continuing to consider our manuscript. Please see our specific responses to each reviewer comment below.
###

*Reviewer #1: Dear Editor:

*Sorry for the late response. I read the revised manuscript, the authors addressed most of the minor points, but I am not sure about the novelties that the authors stressed. If I understand it right, the key technical challenges that XYalign is designed to solve come from two aspects: 1) line 84, the PAR is duplicated on X and Y chromosome reference sequences; 2) the X- and Y-linked sequences maybe similar to each other to cause mis-mapping.

###
The purpose of our paper is not to focus specifically on the PARs, but rather homologous sequences across the entire length of the sex chromosomes. We only highlighted PARs to document a well-known special case of our problem, where we already know sequences are identical. To clarify this point, we have refocused this part of the introduction text, which now reads (lines 77-89):

“This shared origin and complex history characteristic of sex chromosomes lead to unique challenges for genome assembly and analysis, including large blocks of homologous sequence between the sex chromosomes—called gametologous sequence—that we hypothesize can lead to the mismapping of reads between the sex chromosomes. Best known of these gametologous sequences are pseudoautosomal regions (PARs; of which humans have two: PAR1 and PAR2), found in many species—regions identical in sequence between the two sex chromosomes that pair and recombine during meiosis in males [10–13]. A reference genome that includes the entire sequence content from both sex chromosomes will thus duplicate gametologous regions and should substantially reduce mapping quality in these regions because most reads will identically map to two regions in the reference assembly. This stands in contrast to autosomal sequence, for which each diploid autosome is represented just once in the reference genome.”
###

*Such issues emerge when one aligns reads to the nearly-complete genome sequence of human, including both X and Y chromosomes. However, how often do people include both X and Y chromosomes as their reference?

###
For humans, standard practice is to use the entire human reference genome, which includes with both X and Y chromosomes across major sources (GATK Resource Bundle, UCSC Genome Browser, Ensembl, 1000 Genomes, etc.). This is also true of major human datasets, such as ExAC (more than 60 thousand human exomes), 1000 Genomes Project, the Genome-Tissue Expression Project (GTEx; 53 tissues from nearly 1000 individuals), The Cancer Genome Atlas (TCGA; tumor-normal samples from approximately 11,000 individuals). The downstream data from these and other major projects are frequently downloaded and analyzed, so any artifacts from mismapped reads on the sex chromosomes will be present in many additional studies.

With the exception of the 1000 Genomes Project, which masked PARs on the Y chromosome but made no other sex chromosome corrections, we have never seen a genomic analysis that identifies or intentionally corrects for broad-scale (i.e., beyond the PARs) mapping problems we describe in this paper. This is from our combined experience with literature on various vertebrate and invertebrate systems, as well as many conversations with colleagues working on other systems (including plants).
###

*By the way, I do not agree with the statement in the introduction 'the sex chromosomes are routinely excluded from genome-wide analyses.' Maybe the authors specifically mean GWAS studies in human, otherwise a long list of the published genomic work studying sex chromosomes as a hotspot for speciation and sexual selection is missed.

###
We have removed this phrase. This section now reads (lines 89-93):
“The technical challenges presented by the biological realities of the sex chromosomes might lead to erroneous genotype calls. This is unfortunate because the sex chromosomes contribute to phenotype and disease etiology (e.g., [14]) and are useful in population genetic inference of demography and patterns of natural selection [15–19].”
###

*Since human Y chromosome contains very few genes and is very repetitive in sequences, most GWAS studies would only contain the X chromosome as their reference, if they include any sex chromosomes. But the authors may know such papers which actually do that and they need to provide references. Even they actually contain both, then simply mapping the reads separately to an XX genome and a Y chromosome would solve the first issue.

###
We reiterate our point above that we have never encountered any work where researchers selectively choose parts of the reference to include and exclude in this way.

However, in response to this comment, we want to highlight that most GWAS are conducted with SNP data generated from microarrays and therefore would not be mapping to a reference genome. While it might be possible that the homology issue we describe in this paper could affect the success of these experiments, that is well beyond the scope of this study. In addition, contrary to this comment, the two

major microarrays used today, the Affymetrix 6.0 and Illumina Omni2.5, target both the X and Y chromosomes.

For GWAS studies using next-generation sequencing reads, the problem and solution we describe in the paper apply. For this we again reiterate our point above that we have never encountered any work where researchers selectively choose parts of the reference to include and exclude in this way. We agree that mapping XX individuals to a genome with the Y chromosome masked would solve many mapping issues—this is exactly what we describe for the first time in this paper. We, however, disagree about "mapping reads separately to…a Y chromosome". As we discuss in the paper, homology works in both directions, so if the X chromosome is masked for an XY individual, reads from the X chromosome will map to the Y. We instead advocate for a local masking—after mapping—strategy (lines 463-474):

"However, homology is unavoidable for individuals of the heterogametic sex (i.e., XY or ZW) because both sex chromosomes are required in the reference assembly for mapping. In this case, a more local masking or filtering approach is likely the most promising option. For studies investigating specific variants, for which false negatives are preferable to false positives, we suggest strict variant filtering that includes high thresholds for mapping quality (e.g., thresholds of 55 or higher are required to eliminate the effects of homology in the XTR). However, for studies investigating invariant sites as well (e.g., measures of genetic diversity require information from all monomorphic and polymorphic sites), we recommend filtering entire regions based on, at the very least, mapping and depth metrics. These masks are output by the BAM_ANALYSIS module in XYalign, and for this use, we recommend using small windows (e.g, 1 kb to 5 kb) and exploring a variety of depths."
###

*The authors pointed out other example genomes other than human which maybe also affected by the PAR: human, chimpanzee, rhesus macaque, gorilla, mouse, rat, chicken, Drosophila. There are several problems here, first most of them are all assembled by exhaustive Sanger sequencing, with abundant physical mapping information allowing the connection of PAR with X/Y divergent regions. While over 90% of all currently published genomes, or genomes to be published are produced by the second- or third- generation sequencing, including Vertebrate Genomes Project that the author mentioned. Do these genomes have a case where PAR is duplicated on both X and Y chromosomes? A small mistake with the statement is, among the examples, Drosophila does not have PAR sequences and should have no problem at cross-mapping issues between X- and Y-linked gametologs. I am also not sure if gorilla and rat have a Y chromosome sequence assembled including the PAR as duplicated comparing to the X? For the second cross-mapping issue between the gametologs, the authors used the XTR region as the example. This again can be solved by mapping the XX genome and Y genome separately. It is also important that similar regions in other species, i.e., duplicated regions between X and Y chromosomes besides PAR, that show over 95% of sequence identity have been reported, so that the software can be applied to.

###
As we mention in our response above and in our response with our previous resubmission, the effect we describe and with XYalign's utility are not limited to PARs. Figures 1 and 2 and Table 1 illustrate that the problem we describe, though pronounced in PARs, is found across the entire length of the sex chromosomes. In this section we are not discussing PARs, we are simply listing species for which both X and Y chromosomes are available. These species, given the broad homology issues across the sex chromosomes, are most likely to be affected by the problem we describe whether or not PARs are present.

We are confused about the repeated suggestion to map to the "XX genome and Y genome separately", as this is exactly what we propose, for the first time, in our manuscript. However, as we discuss in the manuscript, there are additional considerations—i.e., it's not enough to simply map to different places. Within XYalign, our masking approach, along with some other strategic bioinformatic decisions, allows BAM files to go into variant calling steps with identical sequence header information. Simply mapping to different references will lead to header clashes in the BAM files which cause errors in downstream tools (e.g., GATK). Also, as mentioned above and in our manuscript, XY (and ZW) individuals have the issue of reads mismapping in both directions, so we recommend a local masking approach.
###

*The authors stated several novelties: 1) no one has evaluated 'the effect of sex chromosome homology on downstream analyses'. This is because most species' sex chromosomes share very little homology. And the regions where they share high similarities, in most of the cases, only appear on one sex

chromosome.

###
This is a common misconception and discussed in some detail under “Myth 3” in Batchtrog et al. (2014; PLOS Biology 12(7): e1001899). Across the tree of life, sex chromosomes exhibit a wide range of homology ranging from virtually identical, homomorphic sex chromosomes to highly differentiated sex chromosomes.

Further, in this manuscript, we show that even highly differentiated sex chromosomes, like those found in humans (and across placental mammals), have regions of high homology outside of PARs and XTR that are not found on only one chromosome. See Figures 1 and 2 and Table 1.
###

*2) the second novelty, as I have mentioned, because people would simply just map the reads to the X chromosome.

###
This is novel, as it is something we formally propose for the first time in this manuscript. Also, please see responses above for difficulties involved in “simply” mapping reads to one chromosome or the other.
###

*3) I don't think there is anything unexpected or new from studying the effect of coverage of sequencing on inferring sex, unless a minimum coverage of allowing accurately inferring the sex-linked sequences is reported. As how different sequencing coverage would affect variant calling is clearly known from all previous studies.

###
In our manuscript, we argued that read balance, not depth of coverage, was the more interesting and striking way to determine sex (lines 381-383):

“In our analyses, the most striking measure for assessing an individual’s sex chromosome complement was the distribution of read balances across a chromosome (Figure 4).”

We do discuss issues with establishing a minimum threshold in the manuscript (lines 408-415):

“Across datasets, we observed variation in relative depth of the X and Y chromosomes in XX and XY individuals, particularly among different sequencing strategies: exome, low-coverage whole-genome, and high-coverage whole-genome sequencing (Figure 5A). However, within datasets, XX and XY individuals were clearly differentiated (Figure 5; Supplemental Figure 5). This pattern suggests that a general threshold for assigning different genetic sexes across a range of organisms and sequencing experiments might be difficult to implement. That being said, within species, some combination of depth, mapping quality, and read balance is likely to be informative.”

We do not discuss sequencing coverage and variant calling at all in the manuscript.
###

*Overall, I think it is important the authors point out such a mapping issue when studying human X and Y chromosome. However, I am not sure about this is needed in other species' sex chromosome studies.

###
As we have discussed in our responses above, this phenomenon extends well beyond the PARs (Figures 1-2, Table 1). Given that sex chromosomes, across taxa, evolve from autosomes, this homology issue is likely to be common across species. At the very least, we expect it to be present across placental mammals, who share the same sex chromosomes as humans. However, given the variation in sex chromosome divergence across the tree of life, we expect this to be an issue to plague many groups.
###

*Reviewer #2:
*The authors have addressed my concerns and the methods and results section are much easier to follow. I have some minor comments.

* line 125 and 126: fix parenthesis.

###
We have closed parentheses on both lines (i.e., after [22] and [23]), which now read (lines 235-237):
"…data from one male (HG00512) and one female (HG00513) from the 1000 Genomes Project (Dataset 1; [22]); and (2) 24 high-coverage whole genomes from the 1000 Genomes Project (Dataset 2; [23])."
###

* I think simply putting section "software description" at the start of "Methods" before "Data" might be better. The reason being that several modules of the XYalign pipeline are discussed in "Identifying Effects of Sex Chromosome Homology" and "Inferring Genetic Sex" before they are described in the "software description" section. I don't see that this restructuring requires altering of the text.

###
We have switched the order of those two sections. The final order of sections in the manuscript is: Introduction, Software Description, Methods, Results and Discussion, Conclusion
###

* line 337-338: "…and the pseudoautosomal regions (PAR1 and PAR2) in the reference genome for the XY reference genome, we observed clear improvements in read mapping". The sentence seems to describe observed improvements for both the XX and the XY, but I can only see the improvement of the XX (figure 1-2). From what I can see, no before /after improvement shown for the XY individual. Please include this, or only state improvement observed for the XX individual. Also, the sentence is a bit confusing, maybe change to "…regions (PAR1 and PAR2) in the XY reference genome, we observed…" or reformulate?

###
This sentence now reads (lines 335-337):
"By hard-masking the Y chromosome in the XX reference genome, and the pseudoautosomal regions (PAR1 and PAR2) in the XY reference genome, we observed clear improvements in read mapping for the XX individual (Figures 1-2)."
###

* Line 391: The "strange" allele frequencies. What comes to mind is that this is caused by repetitive regions, e.g., the ampliconic gene families? This could be investigated simply by grouping the genomic coordinates of the frequencies. Do the values from the wide mode 0.05-0.3 come from ampliconic regions?

###
Upon further examination, these frequencies appear to be coming from multiple regions (ampliconic, heterchromatic, and X-transposed). To present these results, we have added Supplemental Table S3 and Supplemental Figures S1-S5 (note that the previous Supplemental Figures S1-S4 are now Supplemental Figures S6-S9). We also updated this section in the text to now read (lines 389-407):

"We observed one exception to this pattern: the Y chromosome exhibited a peak around 0.2 in addition to the one near 1.0 (Figure 4; Supplemental Figure 1). All variants included in analyses met thresholds for depth, site quality, and genotype quality, so quality does not appear to be a driving factor of this pattern. This pattern also remained after genomic windows of low mapping quality and irregular depth were removed. When we parsed variants by Y chromosome region, we discovered that this pattern appears in ampliconic, heterochromatic, and XTR regions, while X-degenerate regions display our expected haploid expectation of a single peak close to 1.0 (Supplemental Figures S2-S5. Moreover, there are fewer sites in the X-degenerate regions than the other bins (Supplemental Table S3). While, taken together, this explains the peak around 0.2 when looking across the entire chromosome (Figure 4; Supplemental Figure 1), we are currently unable to explain the specific factors causing the peak near 0.2 in these regions. Homology is likely playing a role, as both the ampliconic and heterochromatic regions are highly repetitive and the XTR shares homology with the X chromosome. However, more work is required to explore this possibility in more detail and, further, to understand how homology can cause this pattern and lead to what appear to be false positive variants passing all filters. It will additionally be important to determine if similar results are obtained on the W chromosome in ZW systems."
###

* Line 407-409. "…experiment, however, as we did not observe.." remove "as" or complete sentence.

###
We restructured this sentence so it now reads (lines 417-419):

“However, this should be explored in each experiment, as we did not observe this differentiation in the uncorrected 1000 Genomes high-coverage samples (Supplemental Figure S2).”
###

Close
