# Supplementary material for: Identifying, understanding, and correcting technical artifacts on the sex chromosomes in next-generation sequencing data
Source: Gigascience. 2019 Jul 9;8(7):giz074. doi: 10.1093/gigascience/giz074 (PMC6615978; doi:10.1093/gigascience/giz074)
Supplement: giz074_Response_to_Reviewer_Comments_Revision_2 [file giz074_response_to_reviewer_comments_revision_2.pdf]

Author's Response To Reviewer Comments

Close

June 2, 2019

Dear Dr. Hans Zauner and Editors of Gigascience,

We are resubmitting our revised manuscript, GIGA-D-18-00312, titled “Identifying, understanding, and correcting technical artifacts on the sex chromosomes in next-generation sequencing data.” Please note that this title is the same as that of our first and second resubmissions, and slightly different from our original submission, as we now use the word “artifacts” instead of “biases.”

We have addressed all final minor requests and present details on each below.

Please also note that the cover page of the manuscript contains correct, up-to-date author and affiliation information.

In addition, I (T. Webster) will be traveling and unable to respond to emails from June 6 through June 16.

Thank you very much for accepting our manuscript and for all of your help and hard work throughout the review process.

Sincerely,
Timothy H. Webster and Melissa A. Wilson

Editor Comments:

I am pleased to confirm that your manuscript is acceptable for publication in GigaScience. Our data curators had a look over your Zenodo repository and we are pleased that this is equivalent to what we would usually host in our own GigaDB database, so there is no need to duplicate this dataset.

Before we hand over your paper to our production team, please address the following minor requests:

- Please register your software application in the SciCrunch.org database to receive a RRID (Research Resource Identification Initiative ID) number, and include this in your manuscript. This will facilitate tracking, reproducibility and re-use of your tool.

Our SciCrunch RRID, SCR_016661, is now listed in both the “Implementation” and the “Availability of supporting source code and requirements” subsections of the “Software Description” section.

- Please include a section called " Availability of supporting source code and requirements".

We have included the following text at the end of the “Software Description” section:

Availability of Supporting Source Code and Requirements
Project name: XYalign
Project home page: https://github.com/SexChrLab/XYalign
Operating systems: Linux and MacOS
Programming Language: Python
Other requirements: Matplotlib, NumPy, Pandas, PyBedTools, PySam, SciPy, BBTools, BWA, Platypus, Sambamba, and SAMtools.
License: GNU GPL v3
RRID: SCR_016661

-Please also clarify in the manuscript that the Zenodo database also includes the test data used in the article.

Lines 294-296 now read: “We provide templates for all of the analyses described above in the Supplementary Methods. We further provide links to data and Snakemake [51] workflows for all assembly and analysis steps on Github [38] and Zenodo [52].”

Close
