# Supplementary material for: Identifying, understanding, and correcting technical artifacts on the sex chromosomes in next-generation sequencing data
Source: Gigascience. 2019 Jul 9;8(7):giz074. doi: 10.1093/gigascience/giz074 (PMC6615978; doi:10.1093/gigascience/giz074)
Supplement: giz074_Reviewer_1_Report_Original_Submission — Qi Zhou -- 9/1/2018 Reviewed [file giz074_reviewer_1_report_original_submission.pdf]

Reviewer Report

Title: Identifying, understanding, and correcting technical artifacts on the sex chromosomes in next-generation sequencing data

Version: Original Submission **Date:** 9/1/2018

Reviewer name: Qi Zhou

Reviewer Comments to Author:

This paper presents a tool to deal with sex chromosomes, which are distinct from autosomes as the X- and Y- linked alleles (called gametologs) are usually very divergent from each other comparing to the two alleles of autosomes. This tool has two usages: 1) mask the already identified X or Y chromosome sequences for later more accurate calling of variants and coverage data 2) produce metrics and plots for inferring the X- or Y- linkage of unclassified sequences. I acknowledge the importance of the treatment of 1) for downstream analyses of variant calling. However, the scientific or technical novelty presented by this work, and also the scope of application are limited. Both 1) and 2) can be accomplished by all other available routine bioinformatic softwares. For 1), it needs a well-assembled genome like that of human, with X and Y sequences and also their PARs separately assembled as one sequence per chromosome. This is very rare for most species, and also PAR sequences are usually collapsed as one sequence instead of two in these species. For 2), the difficulty usually lies in the 'long-tail' problem, i.e., unclassified scaffold sequences that do not show an expected female vs. male ratio of depth as 0.5 for the X, and as approaching 0 for the Y. This tool does not offer new statistical methods to discriminate scaffolds whose coverage fall in between expectations of sex chromosome and autosome, but just provide their distribution. I have some detailed comments below:
1) page 5, please clarify what does 'secondary and supplementary read mapping' here mean?
2) page 9, I am not sure if we should call these patterns 'artifacts' as they are clearly biologically meaningful. For example, a reduction of MAPQ for the X-transposed region.
3) In figure 1, the depths of PAR, ampliconic regions, are reduced comparing to the rest of the X chromosome, in both A and B plot. It should be clarified, as they are reduced for different reasons. I assume that for PAR, it is because half of the reads were mapped to the other sex chromosome, while for ampliconic regions, it was affected by less uniquely mapped reads comparing to other regions?
4) the MAPQ value of figure 2A is approaching 0, any explanations?
5)page 13, 'the Y chromosome of the XX individual', please clarify what do you mean here?
6) page 15, can you please check where are the reads of 0.2 peak coming from on the Y?

Level of Interest

Please indicate how interesting you found the manuscript: Choose an item.

Quality of Written English

Please indicate the quality of language in the manuscript: Choose an item.

Declaration of Competing Interests

Please complete a declaration of competing interests, considering the following questions:

- Have you in the past five years received reimbursements, fees, funding, or salary from an organisation that may in any way gain or lose financially from the publication of this manuscript, either now or in the future?
- Do you hold any stocks or shares in an organisation that may in any way gain or lose financially from the publication of this manuscript, either now or in the future?
- Do you hold or are you currently applying for any patents relating to the content of the manuscript?
- Have you received reimbursements, fees, funding, or salary from an organization that holds or has applied for patents relating to the content of the manuscript?
- Do you have any other financial competing interests?
- Do you have any non-financial competing interests in relation to this paper?

If you can answer no to all of the above, write 'I declare that I have no competing interests' below. If your reply is yes to any, please give details below.

I declare that I have no competing interests

I agree to the open peer review policy of the journal. I understand that my name will be included on my report to the authors and, if the manuscript is accepted for publication, my named report including any attachments I upload will be posted on the website along with the authors' responses. I agree for my report to be made available under an Open Access Creative Commons CC-BY license (http://creativecommons.org/licenses/by/4.0/). I understand that any comments which I do not wish to be included in my named report can be included as confidential comments to the editors, which will not be published.

I agree to the open peer review policy of the journal

To further support our reviewers, we have joined with Publons, where you can gain additional credit to further highlight your hard work (see: https://publons.com/journal/530/gigascience). On publication of this paper, your review will be automatically added to Publons, you can then choose whether or not to claim your Publons credit. I understand this statement.

Yes Choose an item.
