# Supplementary material for: Identifying, understanding, and correcting technical artifacts on the sex chromosomes in next-generation sequencing data
Source: Gigascience. 2019 Jul 9;8(7):giz074. doi: 10.1093/gigascience/giz074 (PMC6615978; doi:10.1093/gigascience/giz074)
Supplement: giz074_Reviewer_1_Report_Revision_1 — Qi Zhou -- 12/18/2018 Reviewed [file giz074_reviewer_1_report_revision_1.pdf]

**Reviewer Report**

**Title: Identifying, understanding, and correcting technical artifacts on the sex chromosomes in next-generation sequencing data**

**Version: Revision 1** **Date:** 12/18/2018

**Reviewer name: Qi Zhou**

**Reviewer Comments to Author:**

Dear Editor:
Sorry for the late response. I read the revised manuscript, the authors addressed most of the minor points, but I am not sure about the novelties that the authors stressed. If I understand it right, the key technical challenges that XYalign is designed to solve come from two aspects: 1) line 84, the PAR is duplicated on X and Y chromosome reference sequences; 2) the X- and Y-linked sequences maybe similar to each other to cause mis-mapping. Such issues emerge when one aligns reads to the nearly-complete genome sequence of human, including both X and Y chromosomes. However, how often do people include both X and Y chromosomes as their reference? By the way, I do not agree with the statement in the introduction 'the sex chromosomes are routinely excluded from genome-wide analyses.' Maybe the authors specifically mean GWAS studies in human, otherwise a long list of the published genomic work studying sex chromosomes as a hotspot for speciation and sexual selection is missed. Since human Y chromosome contains very few genes and is very repetitive in sequences, most GWAS studies would only contain the X chromosome as their reference, if they include any sex chromosomes. But the authors may know such papers which actually do that and they need to provide references. Even they actually contain both, then simply mapping the reads separately to an XX genome and a Y chromosome would solve the first issue. The authors pointed out other example genomes other than human which maybe also affected by the PAR: human, chimpanzee, rhesus macaque, gorilla, mouse, rat, chicken, Drosophila. There are several problems here, first most of them are all assembled by exhaustive Sanger sequencing, with abundant physical mapping information allowing the connection of PAR with X/Y divergent regions. While over 90% of all currently published genomes, or genomes to be published are produced by the second- or third- generation sequencing, including Vertebrate Genomes Project that the author mentioned. Do these genomes have a case where PAR is duplicated on both X and Y chromosomes? A small mistake with the statement is, among the examples, Drosophila does not have PAR sequences and should have no problem at cross-mapping issues between X- and Y-linked gametologs. I am also not sure if gorilla and rat have a Y chromosome sequence assembled including the PAR as duplicated comparing to the X? For the second cross-mapping issue between the gametologs, the authors used the XTR region as the example. This again can be solved by mapping the XX genome and Y genome separately. It is also important that similar regions in other species, i.e., duplicated regions between X and Y chromosomes besides PAR, that show over 95% of sequence identity have been reported, so that the software can be applied to.
The authors stated several novelties: 1) no one has evaluated 'the effect of sex chromosome homology on downstream analyses'. This is because most species' sex chromosomes share very little homology.

And the regions where they share high similarities, in most of the cases, only appear on one sex chromosome. 2) the second novelty, as I have mentioned, because people would simply just map the reads to the X chromosome. 3) I don't think there is anything unexpected or new from studying the effect of coverage of sequencing on inferring sex, unless a minimum coverage of allowing accurately inferring the sex-linked sequences is reported. As how different sequencing coverage would affect variant calling is clearly known from all previous studies. Overall, I think it is important the authors point out such a mapping issue when studying human X and Y chromosome. However, I am not sure about this is needed in other species' sex chromosome studies.

**Level of Interest**

Please indicate how interesting you found the manuscript: Choose an item.

**Quality of Written English**

Please indicate the quality of language in the manuscript: Choose an item.

**Declaration of Competing Interests**

Please complete a declaration of competing interests, considering the following questions:

- Have you in the past five years received reimbursements, fees, funding, or salary from an organisation that may in any way gain or lose financially from the publication of this manuscript, either now or in the future?
- Do you hold any stocks or shares in an organisation that may in any way gain or lose financially from the publication of this manuscript, either now or in the future?
- Do you hold or are you currently applying for any patents relating to the content of the manuscript?
- Have you received reimbursements, fees, funding, or salary from an organization that holds or has applied for patents relating to the content of the manuscript?
- Do you have any other financial competing interests?
- Do you have any non-financial competing interests in relation to this paper?

If you can answer no to all of the above, write 'I declare that I have no competing interests' below. If your reply is yes to any, please give details below.

I declare that I have no competing interests

I agree to the open peer review policy of the journal. I understand that my name will be included on my report to the authors and, if the manuscript is accepted for publication, my named report including any attachments I upload will be posted on the website along with the authors' responses. I agree for my report to be made available under an Open Access Creative Commons CC-BY license

(http://creativecommons.org/licenses/by/4.0/). I understand that any comments which I do not wish to be included in my named report can be included as confidential comments to the editors, which will not be published.

I agree to the open peer review policy of the journal

To further support our reviewers, we have joined with Publons, where you can gain additional credit to further highlight your hard work (see: https://publons.com/journal/530/gigascience). On publication of this paper, your review will be automatically added to Publons, you can then choose whether or not to claim your Publons credit. I understand this statement.

Yes Choose an item.
