# Supplementary material for: Identifying, understanding, and correcting technical artifacts on the sex chromosomes in next-generation sequencing data
Source: Gigascience. 2019 Jul 9;8(7):giz074. doi: 10.1093/gigascience/giz074 (PMC6615978; doi:10.1093/gigascience/giz074)
Supplement: giz074_Reviewer_2_Report_Original_Submission — Kristoffer Sahlin -- 9/3/2018 Reviewed [file giz074_reviewer_2_report_original_submission.pdf]

Reviewer Report

Title: Identifying, understanding, and correcting technical artifacts on the sex chromosomes in next-generation sequencing data

Version: Original Submission **Date:** 9/3/2018

Reviewer name: Kristoffer Sahlin

Reviewer Comments to Author:

SUMMARY
The authors describes a software XYalign that, in summary, aims to correct misalignments of reads originating from the sex chromosomes. The authors shows that incorrect alignments on the sex chromosomes is common in certain regions, and they occur due to the high sequence similarity between the sex chromosomes, together with the fact that the reference genome does not reflect the structure of the individual (either XX or XY). Their results also indicates that improving the alignments helps downstream variant calling on the sex chromosomes.
XYalign seems to solve a practical problem that the authors encountered, and the software is well documented in terms of installation and user instructions. The introduction gives a clear description of what the aim with the paper. I however find the method and result sections difficult to follow. My assumption is that this is because method descriptions, user recommendations, general notes, and cross referencing other parts of the paper, are interleaved in the text. There are also a lot of parameter settings and command lines given in between text that, in my opinion, could be moved to supplementary to improve the flow of the text. Specific comments below.
MAJOR
* Methods section
- The methods section needs a more formal writing that only focuses on describing the methods. This will help readability and also allow assessment of the methods and to adress any concerns in the analysis. I suggest providing either with pseudocode or text that minimally describe, in detail, the _input_, _workflow_, and _output_ for each of the six modules. This should be presented without including any comments about alternative use cases, comparison to other modules, and other notes (see e.g. "CHROM_STATS" and "CHARACTERIZE_SEX_CHROMOSOMES"). In my opinion, such notes should come after the method is presented.
- After "ANALYZE_BAM" comes a description of "XYalign" (the main program), and the text jumps back to "CHARACTERIZE_SEX_CHROMOSOMES". It makes it difficult to follow the structure.
- Most of the "use cases" section should be moved to supplementary to improve readability. The first paragraph in this section seems to describe the datasets, and could be labelled datasets, and then a quick summary of what was analyzed could be given without providing command lines and text describing specific argument settings.
* Result section
- Which are the datasets presented in the figures 1-4 and table 1 in the results section? Is it averages

across 24 individuals, or only one individual? Provide information if it's Dataset 1 or 2 that is being used.
- If dataset 1 and 2 are being separately analyzed, maybe split the results section up into the two different use cases to clarify and separate the message of the separate analysis.
- Dataset 1: What is the reason behind using exome, low-coverage, and deep coverage data of two individuals? Is it to show that XYalign can work with all these datasets? Which data does is most suitable for XYalign? I see a couple of sentences about this in "inferring genetic sex" but please sharpen the message.
- Table 1. False positives and true negatives are used as labels of the variant calls. This is an overstatement unless the calls are actually validated. If no such additional validation is performed, I suggest to not use this labelling. Either provide an external evaluation of (at least a larger subset of) the calls to give evidence that these are in fact true positives/false negatives, or use other labels, e.g. the actual data presented: "variant calls present before/after masking".
- Under my assumption that Table 1 is data from one individual, I think the manuscript would be significantly strengthened if the authors, found a way to present the results for the 24 individuals combined. For example, maybe variant calls could be compared across individuals e.g. in terms of coordinates. This would be one example of an attempt of validation of the variant calls (related to previous point with false and true calls).
MINOR:
* Provide reason to: "If pseudoautosomal regions (PARs) are present on both sex chromosome sequences in the reference, we strongly suggest masking the PARs on the Y chromosome, allowing reads from these regions to map exclusively to the X chromosome in XY individuals." If two copies are present (onion X and one on Y), wouldn't it be better to keep two copies when mapping to avoid coverage bias? Maybe I'm misinterpreting something here.

Level of Interest

Please indicate how interesting you found the manuscript: Choose an item.

Quality of Written English

Please indicate the quality of language in the manuscript: Choose an item.

Declaration of Competing Interests

Please complete a declaration of competing interests, considering the following questions:

- Have you in the past five years received reimbursements, fees, funding, or salary from an organisation that may in any way gain or lose financially from the publication of this manuscript, either now or in the future?
- Do you hold any stocks or shares in an organisation that may in any way gain or lose financially from the publication of this manuscript, either now or in the future?

- Do you hold or are you currently applying for any patents relating to the content of the manuscript?
- Have you received reimbursements, fees, funding, or salary from an organization that holds or has applied for patents relating to the content of the manuscript?
- Do you have any other financial competing interests?
- Do you have any non-financial competing interests in relation to this paper?

If you can answer no to all of the above, write 'I declare that I have no competing interests' below. If your reply is yes to any, please give details below.

I declare that I have no competing interests

I agree to the open peer review policy of the journal. I understand that my name will be included on my report to the authors and, if the manuscript is accepted for publication, my named report including any attachments I upload will be posted on the website along with the authors' responses. I agree for my report to be made available under an Open Access Creative Commons CC-BY license (http://creativecommons.org/licenses/by/4.0/). I understand that any comments which I do not wish to be included in my named report can be included as confidential comments to the editors, which will not be published.

I agree to the open peer review policy of the journal

To further support our reviewers, we have joined with Publons, where you can gain additional credit to further highlight your hard work (see: https://publons.com/journal/530/gigascience). On publication of this paper, your review will be automatically added to Publons, you can then choose whether or not to claim your Publons credit. I understand this statement.

Yes Choose an item.
