# Supplementary material for: Identifying, understanding, and correcting technical artifacts on the sex chromosomes in next-generation sequencing data
Source: Gigascience. 2019 Jul 9;8(7):giz074. doi: 10.1093/gigascience/giz074 (PMC6615978; doi:10.1093/gigascience/giz074)
Supplement: giz074_Reviewer_2_Report_Revision_1 — Kristoffer Sahlin -- 12/13/2018 Reviewed [file giz074_reviewer_2_report_revision_1.pdf]

Reviewer Report

Title: Identifying, understanding, and correcting technical artifacts on the sex chromosomes in next-generation sequencing data

Version: Revision 1 **Date:** 12/13/2018

Reviewer name: Kristoffer Sahlin

Reviewer Comments to Author:

The authors have addressed my concerns and the methods and results section are much easier to follow. I have some minor comments.
* line 125 and 126: fix parenthesis.
* I think simply putting section "software description" at the start of "Methods" before "Data" might be better. The reason being that several modules of the XYalign pipeline are discussed in "Identifying Effects of Sex Chromosome Homology" and "Inferring Genetic Sex" before they are described in the "software description" section. I don't see that this restructuring requires altering of the text.
* line 337-338: "…and the pseudoautosomal regions (PAR1 and PAR2) in the reference genome for the XY reference genome, we observed clear improvements in read mapping". The sentence seems to describe observed improvements for both the XX and the XY, but I can only see the improvement of the XX (figure 1-2). From what I can see, no before /after improvement shown for the XY individual. Please include this, or only state improvement observed for the XX individual. Also, the sentence is a bit confusing, maybe change to "…regions (PAR1 and PAR2) in the XY reference genome, we observed…" or reformulate?
* Line 391: The "strange" allele frequencies. What comes to mind is that this is caused by repetitive regions, e.g., the ampliconic gene families? This could be investigated simply by grouping the genomic coordinates of the frequencies. Do the values from the wide mode 0.05-0.3 come from ampliconic regions?
* Line 407-409. "…experiment, however, as we did not observe.." remove "as" or complete sentence.

Level of Interest

Please indicate how interesting you found the manuscript: Choose an item.

Quality of Written English

Please indicate the quality of language in the manuscript: Choose an item.

Declaration of Competing Interests

Please complete a declaration of competing interests, considering the following questions:

- Have you in the past five years received reimbursements, fees, funding, or salary from an organisation that may in any way gain or lose financially from the publication of this manuscript, either now or in the future?
- Do you hold any stocks or shares in an organisation that may in any way gain or lose financially from the publication of this manuscript, either now or in the future?
- Do you hold or are you currently applying for any patents relating to the content of the manuscript?
- Have you received reimbursements, fees, funding, or salary from an organization that holds or has applied for patents relating to the content of the manuscript?
- Do you have any other financial competing interests?
- Do you have any non-financial competing interests in relation to this paper?

If you can answer no to all of the above, write 'I declare that I have no competing interests' below. If your reply is yes to any, please give details below.

I declare that I have no competing interests

I agree to the open peer review policy of the journal. I understand that my name will be included on my report to the authors and, if the manuscript is accepted for publication, my named report including any attachments I upload will be posted on the website along with the authors' responses. I agree for my report to be made available under an Open Access Creative Commons CC-BY license (http://creativecommons.org/licenses/by/4.0/). I understand that any comments which I do not wish to be included in my named report can be included as confidential comments to the editors, which will not be published.

I agree to the open peer review policy of the journal

To further support our reviewers, we have joined with Publons, where you can gain additional credit to further highlight your hard work (see: https://publons.com/journal/530/gigascience). On publication of this paper, your review will be automatically added to Publons, you can then choose whether or not to claim your Publons credit. I understand this statement.

Yes Choose an item.
